# Plasma protein changes reflect colorectal cancer development and associated inflammation

**DOI:** 10.3389/fonc.2023.1158261

**Published:** 2023-05-09

**Authors:** Víctor Urbiola-Salvador, Agnieszka Jabłońska, Dominika Miroszewska, Qianru Huang, Katarzyna Duzowska, Kinga Drężek-Chyła, Marek Zdrenka, Ewa Śrutek, Łukasz Szylberg, Michał Jankowski, Dariusz Bała, Wojciech Zegarski, Tomasz Nowikiewicz, Wojciech Makarewicz, Agnieszka Adamczyk, Aleksandra Ambicka, Marcin Przewoźnik, Agnieszka Harazin-Lechowicz, Janusz Ryś, Natalia Filipowicz, Arkadiusz Piotrowski, Jan P. Dumanski, Bin Li, Zhi Chen

**Affiliations:** ^1^ Intercollegiate Faculty of Biotechnology of University of Gdańsk and Medical University of Gdańsk, University of Gdańsk, Gdańsk, Poland; ^2^ Center for Immune-Related Diseases at Shanghai Institute of Immunology, Department of Respiratory and Critical Care Medicine of Ruijin Hospital, Department of Immunology and Microbiology, Shanghai Jiao Tong University School of Medicine, Shanghai, China; ^3^ 3P-Medicine Laboratory, Medical University of Gdańsk, Gdańsk, Poland; ^4^ Department of Tumor Pathology and Pathomorphology, Oncology Center−Prof. Franciszek Łukaszczyk Memorial Hospital, Bydgoszcz, Poland; ^5^ Department of Obstetrics, Gynaecology and Oncology, Collegium Medicum in Bydgoszcz, Nicolaus Copernicus University in Torun, Bydgoszcz, Poland; ^6^ Surgical Oncology, Ludwik Rydygier’s Collegium Medicum in Bydgoszcz, Nicolaus Copernicus University in ToruńSurgical Oncology, Ludwik Rydygier’s Collegium Medicum in Bydgoszcz, Nicolaus Copernicus University in Toruń, Toruń, Poland; ^7^ Department of Surgical Oncology, Oncology Center−Prof. Franciszek Łukaszczyk Memorial Hospital, Bydgoszcz, Poland; ^8^ Department of Breast Cancer and Reconstructive Surgery, Oncology Center−Prof. Franciszek Łukaszczyk Memorial Hospital, Bydgoszcz, Poland; ^9^ Clinic of General and Oncological Surgery, Specialist Hospital of Kościerzyna, Kościerzyna, Poland; ^10^ Department of Tumor Pathology, Maria Skłodowska-Curie National Research Institute of Oncology, Kraków, Poland; ^11^ Department of Immunology, Genetics and Pathology and Science for Life Laboratory, Uppsala University, Uppsala, Sweden; ^12^ Department of Biology and Pharmaceutical Botany, Medical University of Gdańsk, Gdańsk, Poland; ^13^ Faculty of Biochemistry and Molecular Medicine, University of Oulu, Oulu, Finland

**Keywords:** biomarker, colorectal cancer, plasma proteomics, proximity extension assay, inflammation, early detection, cytokines, prognosis

## Abstract

**Introduction:**

Colorectal cancer (CRC) is the third most common malignancy and the second leading cause of death worldwide. Efficient non-invasive blood-based biomarkers for CRC early detection and prognosis are urgently needed.

**Methods:**

To identify novel potential plasma biomarkers, we applied a proximity extension assay (PEA), an antibody-based proteomics strategy to quantify the abundance of plasma proteins in CRC development and cancer-associated inflammation from few μL of plasma sample.

**Results:**

Among the 690 quantified proteins, levels of 202 plasma proteins were significantly changed in CRC patients compared to age-and-sex-matched healthy subjects. We identified novel protein changes involved in Th17 activity, oncogenic pathways, and cancer-related inflammation with potential implications in the CRC diagnosis. Moreover, the interferon γ (IFNG), interleukin (IL) 32, and IL17C were identified as associated with the early stages of CRC, whereas lysophosphatidic acid phosphatase type 6 (ACP6), Fms-related tyrosine kinase 4 (FLT4), and MANSC domain-containing protein 1 (MANSC1) were correlated with the late-stages of CRC.

**Discussion:**

Further study to characterize the newly identified plasma protein changes from larger cohorts will facilitate the identification of potential novel diagnostic, prognostic biomarkers for CRC.

## 1 Introduction

Colorectal cancer (CRC) is the third most common malignancy and the second most lethal cancer, causing 935,000 cancer-related deaths in 2020 (1). CRC prognosis depends mainly on the tumor stage, location, and time of detection. However, despite the huge progress in cancer research, a large number of CRC cases are diagnosed at the advanced stage where cancers are aggressive, malignant, and metastatic (2).

Currently, the most commonly used diagnostic tools for CRC screening and prevention include colonoscopy and flexible sigmoidoscopy, as well as the guaiac-based fecal occult blood test or the immunochemical fecal occult blood test, also known as the fecal immune test (3). These traditional stool-based tests have low sensitivity and specificity (4), while colonoscopy and sigmoidoscopy, despite the high sensitivity, have relatively low compliance, high cost, and are invasive which limits their efficacy in population screening programs (5). Therefore, alternative, non-invasive, and efficient screening strategies to improve the early detection of cancers are urgently needed. Until now, several potential blood-based protein biomarkers for CRC screening and cancer prevention have been reported, including methylated Septin9 (6), extracellular vesicle microRNAs (7), and cell-free circulating DNA (8), but all lack the sensitivity and/or specificity for use as a stand-alone marker.

Advances in proteomic-based technologies in the last decade have expanded the number of candidate biomarkers and led to a better comprehension of the CRC progression as well as the identification and characterization of related molecular signatures. The most recent advancement of Proximity Extension Assay (PEA) allows the quantification of over 3,000 proteins from low amounts of a sample by the combination of DNA-conjugated antibodies and next-generation sequencing (9). Application of the PEA technology has led to the identification of carcinoembryonic antigen (CEA) as one of the best-studied blood-based prognostic biomarkers used in clinical practice (10–12). CEA is expressed in the embryonic endodermal epithelium, colorectal cancer, and other malignancies, such as inflammatory bowel disease (IBD), peptic ulcer, and pancreatitis (13). CEA is a promising plasma biomarker for the detection of CRC with high specificity and sensitivity (12, 14), however, due to the limited organ specificity (15), it is not the best sole biomarker for population-based screening, yet it might be useful in CRC recurrence monitoring (16) and metastasis (17). Currently, the trend in biomarkers discovery is to focus on the biomarker panels rather than on a single-target protein as the broader spectrum of the analysis may help to address the cancer prognosis and detection more precisely.

It was recently reported that two various multimarker panels consisting of five circulating proteins might be used as an efficient tool for the early and late-stage detection of CRC, including advanced adenomas, or in the prediction of overall survival in Germany and Chinese cohorts (18, 19). In a recent study, Harlid et al. (2021) showed that fibroblast growth factor 21 (FGF21) was associated with early, but not late stages of colon cancer, while pancreatic prohormone (PPY) was a promising biomarker for rectal cancer detection (20). However, neither FGF21 nor PPY could be used as stand-alone biomarkers for colon or rectal cancer but might be used as an efficient tool to discriminate between different subtypes of CRC. Therefore, there is an urgent need for the identification of a reliable blood-based biomarker panel that would detect the early stages of CRC as well as assesses the prognosis at the population-based screening.

Both chronic inflammation, such as IBD, and sporadic, cancer-associated inflammation are well-known as key factors in CRC progression and development. Inflammation alters the communication between a variety of cell types, including innate and adaptive immune cells, epithelial cells, and stem cells. These intricate networks of cytokines, growth factors, receptors, and other molecules interaction result in either a tumor-promoting or inhibiting environment (21). Thus, in the development of plasma biomarkers for CRC diagnosis, prognosis, and immunotherapy, the inflammatory status is essential.

The purpose of our study was to identify the protein expression changes in the plasma of CRC patients compared to healthy controls as well as between the early and late stages of CRC and inflammatory status. Therefore, an inflammation panel including 368 proteins was selected to be detected in this study. We hypothesized that CRC development, tumor stage, and inflammation-caused changes in protein level will be reflected in the circulating blood and as such, we would be able to obtain a panel of biomarkers with potential translation into clinics to improve patient care. In this study, we quantified the plasma protein profiles derived from 38 CRC patients and their age- and sex-matched 38 healthy subjects using the PEA technology and protein panels consisting of 368 oncology- and 368 inflammation-related protein biomarker candidates. We quantified 690 proteins, among which 78 differentially expressed proteins (DEPs), were elevated and 124 DEPs were reduced in patients with CRC. We found protein signatures associated with cytokine interactions, oncogenic signaling pathways, exacerbated apoptosis, as well as metabolism reprogramming. Additionally, we determined protein changes linked to cancer-associated inflammation and novel potential prognostic biomarkers associated with tumor stages. Linear regression model analysis revealed that carbonic anhydrase (CA11), a cluster of differentiation 276 (CD276), colony-stimulating factor 3 (CSF3), and interleukin 12 receptor subunit beta 1 (IL12RB1), were positively associated with inflammatory status, whilst amyloid beta precursor protein binding family B member 1 interacting protein (APBB1IP) and C-X-C motif chemokine ligand 6 (CXCL6) were negatively associated. Moreover, linear regression model analysis of tumor stage indicated high plasma levels of Fms-related tyrosine kinase 4 (FLT4), MANSC domain-containing protein 1 (MANSC1), and lysophosphatidic acid (LPA) phosphatase type 6 (ACP6), that could be used as potential prognostic biomarkers for advanced CRC. In contrast, high levels of interferon γ (IFNG), interleukin (IL)32, and IL17C in early CRC stages indicate that these proteins can discriminate between early and late stages, patients. Validation of these identified plasma protein changes with larger cohorts will facilitate the identification of potential novel diagnostic, prognostic biomarkers for CRC.

## 2 Methods

### 2.1 Study cohort

The study was retrospective and consisted of 38 patients who underwent CRC surgery (mean age: 66.7 ± 12.3; 42.1% male) between June 2019 and April 2021 and 38 age- and sex-matched healthy subjects. All CRC patients had a positive colonoscopy and pathology-confirmed malignant neoplasm of the rectum or colon. Among them, 63.2% (24/38) were diagnosed with late-stage CRC (III-IV) according to the Union for International Control TNM classification and 28.9% (11/38) had inflammation according to the pathologist assessment (**Table 1**). Samples collected from CRC patients and healthy subjects were obtained from the 3P–Medicine Laboratory, Medical University of Gdansk (22) and Biobank HARC, Medical University of Lodz, respectively. In order to validate the assay in an independent cohort, serum samples from 41 patients who underwent CRC surgery (mean age: 58.9 ± 10.1; 43.9% male) were obtained from the Bank of Biological Material at Masaryk Memorial Cancer Institute, Czech Republic. Supported by the project BBMRI.cz no. LM2023033. Whole blood samples were collected into sterile BD Vacutainer^®^ K2EDTA tubes during the day of the planned CRC resection, centrifuged, aliquoted plasma and serum, and stored at -80°C until use.

**Table 1:** Clinical characteristics of patients with CRC.

Patient	Age	Sex	Tumor stage	Inflammatory status
P1	49	F	Late	+
P2	77	F	Late	+
P5	71	M	Late	+
P6	77	F	Early	+
P7	85	F	Early	+
P9	72	M	Late	+
P10	62	M	Late	+
P11	56	F	Early	+
P12	80	M	Late	+
P13	38	F	Early	+
P14	89	F	Late	–
P15	73	F	Early	–
P16	76	F	Early	–
P17	42	M	Late	–
P18	61	M	Early	–
P19	62	F	Early	–
P20	76	M	Late	–
P21	56	F	Late	+
P22	50	F	Late	–
P23	42	M	Late	–
P24	53	F	Late	–
P25	75	M	Early	–
P27	60	M	Late	–
P28	63	F	Early	–
P29	67	F	Late	–
P30	83	M	Late	–
P31	73	M	Late	–
P32	63	M	Late	–
P35	73	F	Early	–
P36	64	F	Early	–
P37	68	F	Late	–
P38	61	M	Early	–
P39	80	M	Late	–
P40	77	F	Early	–
P41	73	F	Late	–
P42	63	F	Late	–
P43	79	F	Late	–
P44	67	M	Late	–

F, female; M, male; Early (I and II stages); Late (III and IV stages); -, non-inflammation; +; inflammation.

### 2.2 Protein profiling

Plasma proteins were analyzed using the multiplex PEA technology (Olink^®^ Explore 384-Oncology and -Inflammation panel, Olink Proteomics, Uppsala, Sweden) (**Supplementary Tables 1** and **2**). Briefly, the PEA technology is a dual recognition approach based on matched pairs of oligonucleotide-labeled antibodies that bind to their target proteins. Once the target proteins are bound, the oligonucleotides brought into proximity, hybridize and are detected and quantified by using next-generation sequencing (9, 20). PEA quantifies a large number of proteins (> 3,000) with good precision, using a minimal volume of plasma or serum samples, and without loss of specificity and sensitivity. The protein levels are presented in the normalized protein expression (NPX) values on a log2 scale. A high protein concentration corresponds to a high NPX value. For quality assessment and validation of the PEA technology, the protein level of ACP6 was measured by ELISA, while for CSF3, IFNG, IL6, CXCL9, and CCL23 were determined by using Luminex MAGPIX technology.

### 2.3 Statistical analyses

Almost all statistical analyses were performed in RStudio (version 1.3.1093) using R (version 4.0.3). First, proteins were filtered when the quality control was negative or the calculated NPX values were below the respective protein limit of detection (LOD) in at least 50% of samples from one of the study groups. The remaining NPX values below the LOD were imputed with the respective LOD/√2. Moderated t-test from the R package “limma” (version 3.46.0) was used to test differential protein abundance between CRC patients and healthy subjects. Additional analysis was performed using the general linear model regression approach with analysis of contrasts using the R package “emmeans” (version 1.6.2.1). A general linear model was fitted to the expression of each protein in all CRC patients using tumor stage and inflammation as independent variables, and sex as a confounding factor. The false discovery rate (FDR) was determined using the Benjamini & Hochberg correction. Proteins were considered differentially expressed when FDR adjusted *p*-value < 0.05. The built-in R function cor.test was used to calculate the point-biserial correlation between protein expression and tumor stage or inflammation status, the *p*-value < 0.05 was considered significant. Gene set enrichment analysis with Gene Ontology terms was performed using ClusterProfiler (version 4.6.0), while Kyoto Encyclopedia of Genes and Genomes (KEGG) pathway enrichment analysis *via* active subnetworks from STRING database was conducted using “pathfindR” (version 1.6.3), with FDR < 0.05. “ggplot2” (version 3.3.5) was used for graphics generation, excluding heatmaps that were generated using “ComplexHeatmap” (version 2.6.2). The hierarchical clustering (Euclidean distance) was implemented to visualize the patterns of DEPs among samples after the z-score transformation of NPX values; DEPs were split by k-means clustering. T test was used for the calculation of continuous variables (protein levels) by using GraphPad Prism version 9.0 (GraphPad Software Inc., San Diego, CA, USA).

## 3 Results

### 3.1 CRC development causes cytokine and oncogenic signaling pathway changes in plasma

**Figure 1:**
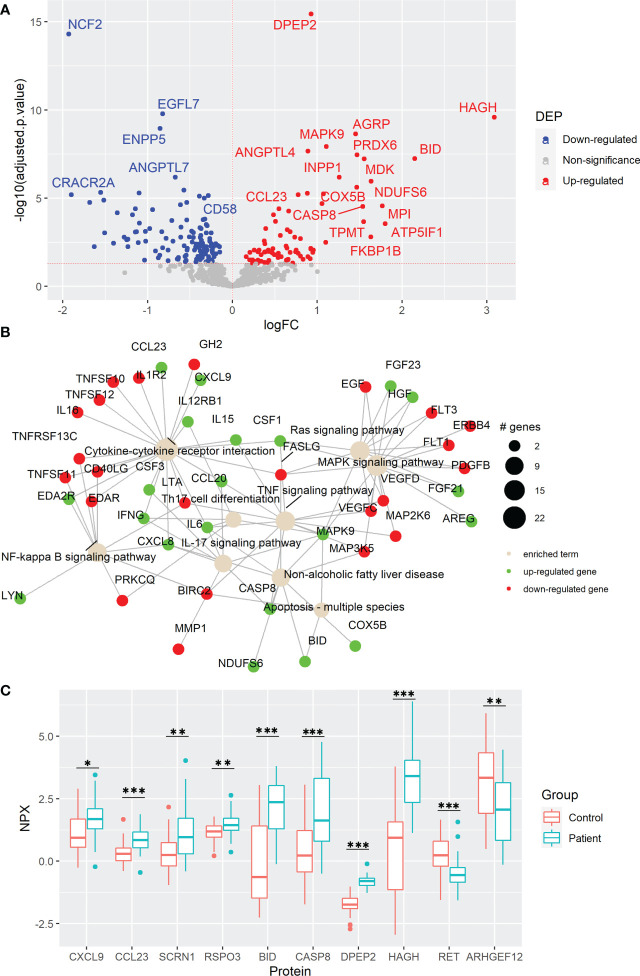
Colorectal cancer (CRC) development causes cytokine and oncogenic signaling pathway changes in plasma. **(A)** Volcano plot of statistical significance against fold-change of proteins between CRC patients and healthy controls. Dots indicate individual proteins and the red and blue dots represent significant up-regulation and down-regulation in patients, respectively. **(B)** Network of KEGG pathway enrichment analysis combined with STRING protein-protein interaction network analysis. Green and red proteins indicate significant up-regulation and down-regulation, respectively. **(C)** Box and whisker plots of selected DEPs not previously reported associated with CRC. * indicates statistically significant with an adjusted p-value < 0.05, ** indicates an adjusted p-value < 0.01, and *** indicates an adjusted p-value < 0.001. DEP, differentially expressed protein; FC, fold change, NPX; normalized protein expression.

To determine the changes in the protein profiles in peripheral blood caused by CRC development, we performed plasma protein analysis by using PEA technology. Out of the total 736 proteins from the Inflammation and Oncology Explore panels, after removing repetitions in the panels and after removal of proteins with low detection rates among the samples, 690 proteins were quantified. Among them, 78 proteins were elevated and 124 were reduced in the 38 CRC patients compared with their age- and sex-matched healthy controls (**Figure 1A**, **Supplementary Figure 1A**, and **Supplementary Table 3**). Of the elevated DEPs, dipeptidase 2 (DPEP2), hydroxyacylglutathione hydrolase (HAGH), and agouti-related neuropeptidase (AGRP) as well as downregulated DEPs as neutrophil cytosolic factor 2 (NCF2), epidermal growth factor-like protein 7 (EGFL7), and ectonucleotide pyrophosphatase/phosphodiesterase family member 5 (ENPP5) were the DEPs with the most statistical difference. In line with previous studies which were carried out with different technologies for protein detection and quantification (17, 23–28), high plasma levels of AGRP, FGF21, midkine (MDK), C-C motif chemokine ligand 20 (CCL20), IL6, and CSF3 as well as reduced ribonucleotide reductase regulatory TP53 inducible subunit M2B (RRM2B) on plasma level of CRC patients were also identified in our study. Importantly, we found novel protein changes including high levels of oncogenic proteins such as R-Spondin 3 (RSPO3) and secernin 1 (SCRN1) as well as low levels of tumor suppressors such as Ret proto-oncogene (RET) and Rho guanine nucleotide exchange factor 12 (ARHGEF12) in CRC patients. These results suggest the association between plasma protein levels and protein expression within the tumor microenvironment (TME).

To investigate the involved pathways and the complex protein-protein interactions among these DEPs, KEGG enrichment analysis *via* active subnetworks was performed. Plasma protein changes were mainly associated with the cytokine-cytokine receptor interaction, including high plasma level of T-cell chemoattracting chemokine CXCL9 and the immune cell chemoattractant CCL23, as well as several signaling pathways including mitogen-activated protein kinase (MAPK), resistance to audiogenic seizures (Ras), tumor necrosis factor (TNF), nuclear factor kappa B (NF-κB), and IL17 signaling pathways (**Figures 1B, C**, and **Supplementary Table 4**). Notably, proteins involved in Th17 cell differentiation were upregulated in CRC patients, suggesting an active role of this T-cell helper subtype in CRC development. Moreover, proteins related to non-fatty liver disease (NAFLD), a disease previously associated with CRC risk (29), were enriched (**Figure 1B** and **Supplementary Tables 3, 4**). At the same time, high levels of apoptosis-associated proteins, caspase-8 (CASP8) and BH3 interacting domain death agonist (BID) were discovered, with BID having the second highest fold change in the comparison (**Figures 1A–C**). To reveal possible mechanisms of cancer development, DEPs were further evaluated by using gene set enrichment analysis. This analysis revealed that the gene ontology terms including oxidative phosphorylation, aerobic respiration, respiratory electron transport chain, and ATP synthesis coupled electron transport in mitochondria were enriched in CRC patients (**Supplementary Figure 1B** and **Supplementary Table 5**). Moreover, other metabolic proteins were highly elevated in CRC patients including HAGH and DPEP2 (**Figure 1C**) which may reflect the metabolism reprogramming due to CRC tumorigenesis, a well-known hallmark of cancer (30).

Next, to distinguish which of the protein changes were a consequence of an altered secretion from a certain type of cells and which were a result of destructed tissues or cells released during CRC tumorigenesis, from the 202 DEPs, 50 proteins were identified in the human blood secretome from Human Protein Atlas, including cytokines that modulate the immune responses within the TME, such as IFNG, IL6, IL15, CCL20, CXCL9, and CCL23 (**Supplementary Table 6**). Some of these cytokines were previously found with high plasma levels in CRC such as pro-inflammatory cytokine IL6 which is also required for Th17 differentiation (26), the pro-inflammatory MDK involved in multiple biological processes (17), and the chemoattractant of B- and T-cells CCL20 (25), whereas the detected IFNG is a well-recognized pro-inflammatory and antitumorigenic protein (31). Interestingly, the elevated plasma levels of the chemoattractants CXCL9 and CCL23 in CRC patients were reported for the first time in our study. These results suggest that plasma protein changes can reflect the variety of altered processes involved in tumorigenesis. Collectively, CRC development causes protein changes in plasma that are linked to several signaling pathways, cytokine interactions of underlying immune responses, and altered metabolism.

### 3.2 Cancer-associated inflammation alters the plasma protein expression in CRC patients

**Figure 2:**
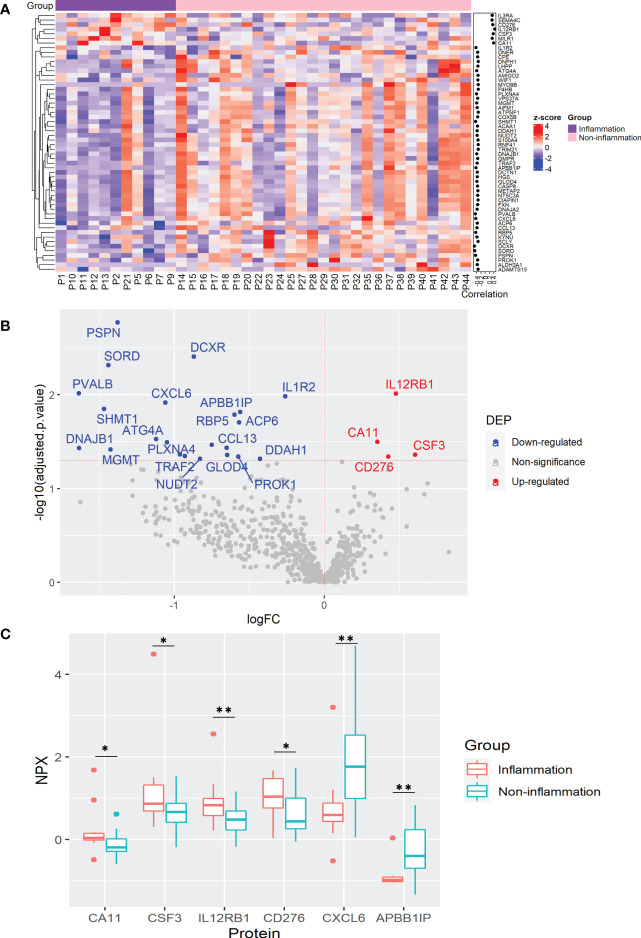
Plasma protein changes induced by cancer-associated inflammation in CRC patients. **(A)** Heatmap of proteins with significant correlation with inflammation status. Protein expression is transformed with a z-score by row normalization and distributed by hierarchical clustering. The correlation coefficients (right) indicate a positive/negative correlation for each protein. **(B)** Volcano plot of statistical significance against fold-change of proteins between CRC patients with inflammation and without inflammation. Dots indicate individual proteins and the red and blue dots represent significant up-regulation and down-regulation in CRC patients with inflammation, respectively. **(C)** Box and whisker plots of selected DEPs not previously associated with cancer-related inflammation in CRC patients. *: adjusted p-value < 0.05, **: adjusted p-value < 0.01.

It is well-known that chronic inflammation may contribute to cancer development. To determine plasma protein changes related to inflammatory status in CRC patients, we analyzed DEPs among patients with and without inflammation (11 and 27 cases, respectively). Correlation analysis revealed 56 proteins significantly correlated with inflammation, among which 7 proteins, CA11, CD276, CSF3, IL3RA, IL12RB1, MILR1, and SEMA4C were positively correlated, while 46 proteins including ACP6, APBB1IP, CXCL6, and dicarbonyl and L-xylulose reductase (DCXR) were correlated negatively (**Figure 2A** and **Supplementary Table 7**). Among them, elevated IL12RB1 and reduced DCXR showed the highest correlation with inflammatory status (**Figure 2A** and **Supplementary Table 7**). To confirm the association between protein expression and inflammation, a linear regression analysis was used to determine the differential expression of these proteins. As a result, 26 DEPs were identified which were significantly correlated in the previous analysis (**Figure 2B** and **Supplementary Table 8**).

KEGG pathway enrichment analysis demonstrated that the DEPs were mainly assigned to cytokine-cytokine interaction, IL17 and Th17 cell differentiation, and Janus kinase (JAK)-signal transducer and activator of transcription (STAT) signaling pathways, as well as pentose and glucuronate conversion (**Supplementary Table 9**). It has been well documented that Th17 cells play an essential role in inflammation *via* the production of pro-inflammatory cytokines IL17A, IL17F, IL22, and IL21. Th17 cell activity is also associated with an increased risk of CRC tumorigenesis (32). Among the DEPs involved in the IL17, Th17 cell differentiation and JAK-STAT signaling pathways, elevated levels of CSF3 and reduced CXCL6 were previously found in the serum of CRC patients (27, 33), while our study also demonstrates their association with cancer-associated inflammation (**Figures 2A–C**). Interestingly, CSF3 is involved in inflammation by inducing bone-marrow neutrophil differentiation and its high levels are related to CRC tumorigenesis (27). Moreover, we report, for the first time, the association of IL12RB1, CA11, CD276, and APBB1IP with cancer-associated inflammation (**Figure 2C**). Accordingly, IL12RB1 and CSF3 were detected with high plasma levels in the whole CRC patients compared with healthy controls (**Figure 1A** and **Supplementary Table 3**). It is worth noting that IL12RB1, CD276, and APBB1IP are involved in cancer surveillance, inhibition of T-cell mediated responses, and T-cell recruitment, respectively (34–36), whereas CA11 may induce proliferation and invasion of gastrointestinal tumors (37) (**Figure 2C**). In summary, these results suggest that inflammation in CRC patients can influence plasmatic protein levels. Furthermore, these proteins may be useful indicators of cancer-associated inflammation that may complicate the outcome of CRC patients.

### 3.3 Determination of potential plasma biomarkers associated with CRC stages

**Figure 3:**
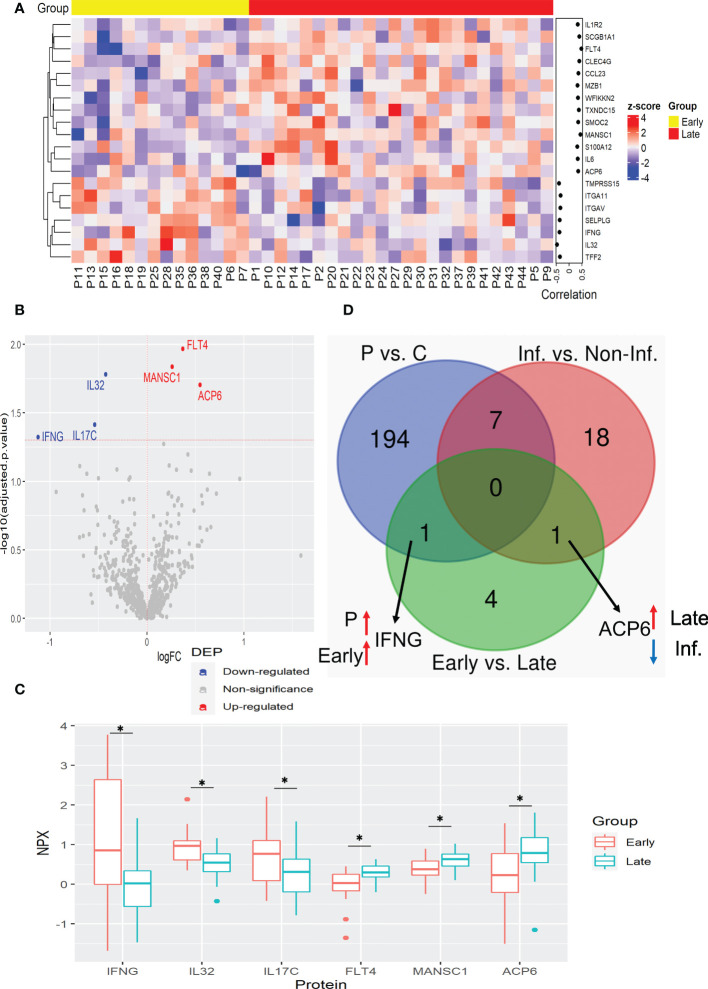
Plasma protein expression differences between early and late stages of CRC. **(A)** Heatmap of proteins with significant correlation with tumor stage. Protein expression is transformed with a z-score by row normalization and distributed by hierarchical clustering. The correlation coefficients (right) indicate a positive/negative correlation for each protein. **(B)** Volcano plot of statistical significance against fold-change of proteins between CRC patients with early tumor stage and with late tumor stage. Dots indicate individual protein and the red and blue dots represent significant up-regulation and down-regulation in CRC patients with late tumor stage, respectively. **(C)** Box and whisker plots of DEPs that are novel potential prognostic biomarkers associated with cancer stages in CRC patients. *: adjusted p-value < 0.05. **(D)** Venn diagram with the differentially expressed proteins for each comparison: CRC patients vs. control, Inflammation vs. Non-inflammation, and Early vs. Late. Black arrows indicate the proteins of interest that are in common between comparisons. Red and blue arrows indicate up-regulation and down-regulation for the specified group, respectively. C, control; Inf., inflammation; Non-Inf., non-inflammation; P, patient.

The main cause of a patient’s death due to CRC is tumor growth and its increased invasiveness, resulting in metastasis. Therefore, it is crucial to find prognostic biomarkers for CRC progression. We determined the plasma protein changes associated with CRC advance by the comparison of patients with early (I and II) and late (III and IV) stages of CRC. The correlation analysis showed that 13 proteins, ACP6, CCL23, C-type lectin domain family 4 member G (CLEC4G), FLT4, IL1R2, IL6, MANSC1, marginal zone B and B1 cell-specific protein (MZB1), S100 calcium-binding protein A12 (S100A12), secretoglobin family 1A member 1 (SCGB1A1), SPARC-related modular calcium-binding protein 2 (SMOC2), thioredoxin domain containing 15 (TXNDC15), and WAP, follistatin/kazal, immunoglobulin, kunitz and netrin domain containing 2 (WFIKKN2) were positively correlated with tumor stage, whereas 7 proteins, including IFNG, IL32, integrin subunit alpha 11 (ITGA11), ITGAV, selectin P ligand (SELPLG), trefoil factor 2 (TFF2), and transmembrane serine protease 15 (TMPRSS15) were correlated negatively (**Figure 3A**). Among them, FLT4 showed the best prognostic performance for late-stage CRC with the highest correlation coefficient (**Figure 3A** and **Supplementary Table 10**). The elevated plasma FLT4, also named Vascular Endothelial Growth Factor Receptor 3 (VEGFR3) in the late stage of CRC may be associated with VEGF-mediated lymphangiogenesis and angiogenesis.

Similarly to the analysis with inflammatory status, the regression analysis resulted in fewer DEPs than correlated proteins. This analysis revealed that ACP6, FLT4, and MANSC1 were elevated in the late stages of CRC, while IL17C, IL32, and IFNG were elevated in the early stages (**Figures 3B, C**, and **Supplementary Table 11**). Notably, the enzyme ACP6 which is involved in phospholipid metabolism by hydrolysis of LPA was negatively associated with inflammatory status, suggesting that ACP6 may play a role in both inflammation and CRC progression (**Figure 3D**).

### 3.4 Validation of identified plasma protein changes with a different cohort

**Figure 4:**
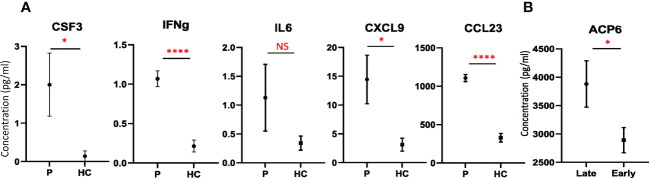
Validation of potential candidate biomarkers. **(A)** Plots with the concentrations of CSF3, IFNG, IL6, CXCL9, and CCL23 in CRC patients (P) and healthy controls (HC) (mean ± SEM) detected by Luminex. **(B)** Plot with the concentrations of ACP6 detected by ELISA in CRC patients with early and late stages, respectively (mean ± SEM). T test was used for statistical analysis. *: p-value < 0.05, ****: p-value < 0.0001, NS: non-significance.

To validate some of the newly identified plasma protein changes in CRC patients, an independent cohort including 41 patients who underwent CRC surgery obtained from the Bank of Biological Material at Masaryk Memorial Cancer Institute, Czech Republic was used. Higher concentrations of IL6 and CSF3 among CRC patients than in healthy volunteers were confirmed in the validation stage of the study (**Figure 4A**). Importantly, increased secretion of IFNG, CXCL9 and CCL23 in the plasma of CRC patients compared to healthy subjects was detected in the validation cohort by Luminex (**Figure 4A**), suggesting that elevated plasma level of IFNG, CXCL9 and CCL23 might be served as a biomarker of CRC. Importantly, similar as detected by PEA (**Figure 3B**), the elevated level of ACP6 in late stage compared with early stage of CRC was confirmed in this cohort as well (**Figure 4B**). Taken together, these results indicate that ACP6 might be a potential prognostic marker for advanced CRC. Notably, MANSC1 and ACP6 have not been previously reported to be associated with CRC development. However, these findings need to be confirmed by using bigger validation cohort.

## 4 Discussion

Cancer, including colorectal cancer, is the leading cause of death worldwide and the most devastating disease as the 21st century begins. Thus, there is an urgent need for the discovery and validation of reliable and efficient non-invasive biomarkers for early CRC detection and prognosis prediction, including biomarkers to detect cancer-associated inflammation. To determine plasma protein changes in CRC patients, by using PEA technology, we quantified 690 proteins, among which 202 were changed compared to healthy subjects.

Among the elevated cytokines in CRC patients, CXCL9 and CCL23 have been identified as novel potential biomarkers. The T-cell chemoattractant *CXCL9* was previously found elevated in CRC tissues compared to normal colon tissues and it was associated with tumor differentiation and invasion, lymph node and distant metastasis, as well as with vascular invasion (38). An enhanced expression of *CXCL9* in cancer tissue than healthy tissue was also observed in the second Chinese study, where *CXCL9* expression levels were associated with tumor stage and survival (39). Importantly, CXCL9 may also recruit T-cells to the TME and exerts antitumor activity (40). The chemokine, CCL23 has been found as a cytokine with both, pro- and anti-cancer properties. It can induce angiogenesis by activating C-C Motif Chemokine Receptor 1 (CCR1) on vascular endothelial cells and increase the proliferation of cancer cells, but also, it can promote immune infiltration (41). However, what type of immune cells and T-cells are attracted to the TME by CCL23 and CXCL9, respectively, requires further studies. A strong elevation of CCL23 protein was noticed in rectal cancer compared to non-rectal cancer consisting of ascending, transverse, and sigmoid colon (42), while *CCL23* expression was not detected in colon adenocarcinoma cells in a second study (43). Interestingly, none of the previous studies reported high CXCL9 and CCL23 levels in the plasma of CRC patients.

Apart from cytokines, plasma levels of other immune-related proteins were changed in CRC patients compared to the healthy controls, such as DPEP2 and Peroxiredoxin 6 (PRDX6), which have not been previously reported as plasma diagnostic biomarkers. The protein expression of DPEP2, a dipeptidase involved in leukotriene metabolism, was recently found as a modulator of macrophage inflammatory responses, protecting mice against Coxsackievirus B3-induced viral myocarditis (44). Interestingly, DPEP1, the paralog of DPEP2 was up-regulated in CRC tissue at mRNA and protein levels and high DPEP1 expression was significantly correlated with cancer stage, location, and poorer prognosis (45), while no association of DPEP2 with CRC has been detected. Similarly, elevated PRDX6, a metabolic enzyme, may modulate inflammation and immune responses through the regulation of antioxidants and reactive oxygen species (46). It was suggested that PRDX6 may promote CRC invasiveness and aggressiveness by inducing an oxidizing TME (47). Importantly, we found two mediators of apoptosis, CASP8 and BID, which presented high plasma levels in CRC patients compared to healthy subjects, with BID having the second highest fold change. Recently, circulating CASP8 was identified with high expression in pre-operative serum samples of prostate cancer (48). BID, belonging to the B-cell lymphoma 2 (BCL-2) family, is a key regulator of apoptosis and a factor associated with CRC initiation and progression (49). It was found that high expression of proapoptotic BID was a predictor of overall survival in patients with CRC, whereas combined expression of BAD and BID was associated with disease-free survival rates and overall survival (50). However, further studies are needed to investigate whether the elevated plasma CASP8 and BID are associated with an exacerbated apoptosis of peripheral blood mononuclear cells (PBMC) among these patients, similarly as in the case of melanoma patients (51). Collectively, the altered cytokines and immune-related proteins suggest an active modulation of the immune system in CRC patients at the systemic level as well as a systemic inflammatory status.

It is well-known that several signaling pathways, such as Ras, NF-κB, and MAPK are altered in CRC patients leading to oncogenesis (52), which was also confirmed in our study at a systemic level. Interestingly, several oncogenic proteins were elevated in plasma, such as SCRN1 and RSPO3, whereas previous studies determine their overexpression in CRC tumor tissue (53, 54). SCRN1 accelerates tumor progression by the regulation of exocytosis of matrix metalloproteinase-2/9 (MMP-2/9) (55), while RSPO3 is an oncogenic driver that causes CRC and extensive crypt hyperplasia, concomitantly stimulating stem cells and supportive niche cells (56). It was found that overexpression of *RSPO2* and *RSPO3* was presented by 4-10% of colon subjects (54) and recurrent R-spondin fusions in colon cancer activate the Wnt signaling and increase the tumorigenesis (57). Additionally, lower plasma levels of potential tumor suppressor proteins, such as RET and ARHGEF12 were detected in CRC patients. RET, is a transmembrane receptor tyrosine kinase and a receptor for the GDNF-family ligands, which downregulation in CRC tissue compared to healthy tissue was noticed (58). CRC patients with somatic RET mutations exhibited a lower incidence of liver metastasis but a higher incidence of peritoneal metastasis and more frequently exhibited mucinous histology (59). On the other hand, a germ-line or somatic RET mutation was linked with more intense and complete angiogenesis in patients with advanced medullary thyroid cancers (60). ARHGEF12, also known as leukemia-associated Rho guanine-nucleotide exchange factor (LARG), is underexpressed in CRC tissue and is associated with reduced cell proliferation and a slower migration rate in cancer cells (61). Moreover, it was found that ARHGEF12 regulates cell adhesion and structure morphogenesis in esophageal squamous cell carcinoma tissues (62) and plays a key role in erythroid regeneration after chemotherapy in acute lymphoblastic leukemia patients (63). These proteins can be potentially used as an oncogenic protein signature for CRC diagnosis in plasma. Apart from oncogenic pathways, NAFLD was also enriched in this cohort. Meta-analyses revealed that NAFLD was associated with an increased risk of gastrointestinal cancers (64) and colon cancers, especially in the right-sided colon (29).

More importantly, our data demonstrated the upregulation of Th17 cell differentiation in CRC patients. Th17 activity has been linked to CRC tumorigenesis and poor prognosis (65). It is well-known that chronic inflammation contributes to cancer development. We identified upregulation of IL12RB1 and CSF3 in Th17 differentiation and IL17 signaling, indicating their participation in CRC-related inflammation. It is worth noticing that CSF3 expression was previously found elevated in the serum of CRC patients (27). An increased gene expression of CSF3 was also observed in CRC tissue from two Consensus Molecular Subtypes (microsatellite instable immune and mesenchymal), where it was associated with regulators (e.g., CXCL5) of invasion (66). IL12RB1, a subunit of the interleukin 12 receptors is associated with tyrosine kinase 2 (TYK2), which plays a pivotal role in immunity to viral infection and cancer surveillance (34). It was found that elevated expression of tumor tissue IL12RB1 was associated with lung cancer progression (67), whereas its correlation with CRC development has not been reported. Moreover, IL12RB1 contributes to both the IL12- and IL23-signaling pathways and is involved in both Th1 and Th17 cell differentiation (68). A carbonic anhydrase, CA11, was also associated with inflammation which overexpression promotes the proliferation and invasion of gastrointestinal tumors without any previous association with CRC in plasma (37). Importantly, the immune checkpoint inhibitor CD276, also called B7-H3 was also linked to inflammation. CD276 was previously reported with high expression in CRC tissue and may contribute to the tumor evasion of T-cell mediated responses (35, 69) and has been already proposed as a target for immunotherapy (70). The overexpression of this immune checkpoint molecule in our study further indicates the importance of this protein in the personalized medicine and immune-checkpoint therapy aspect.

In contrast, the reduced plasma level of CXCL6 and APBB1IP in CRC patients with inflammation was observed in our study. It was recently found that low serum CXCL6 levels were associated with an increased risk of CRC development (33), while CXCL6 expression is not altered in CRC tissue (71). The APBB1IP is a Rap1-binding protein that acts as a regulator of leukocyte recruitment and pathogen clearance through complement-mediated phagocytosis (36). It was shown that expression of APBB1IP was correlated with the prognosis of various cancer types and its upregulation has been demonstrated as associated with increased immune cell infiltration, especially CD8^+^ T cells, natural killer (NK) cells, and immune regulators (36). Bioinformatics analyses revealed that *APBB1IP* may be used as a potential biomarker for osteosarcoma metastasis (72) and suggested its potential role in the evolutionary mechanisms of head and neck squamous cell carcinoma related to inflammation and TME (73). Moreover, cancer-related inflammation may cause the downregulation of APBB1IP decreasing the recruitment of leukocytes to the TME. In this study, for the first time, we reported the association of reduced plasma APBB1IP level with CRC and inflammation, suggesting that APBB1IP could be a potential biomarker for inflammation-associated CRC.

The next two elevated plasma proteins, MANSC1 and ACP6, identified in our study have never been suggested as associated with CRC risk. Expression of bone marrow *MANSC1* was detected in patients with different hematologic malignancies such as acute myeloid leukemia, myelodysplastic syndromes, and primary myelofibrosis, but no significant correlations between the expression of the gene and survival were observed (74). In contrast, an association between high expression of *MANSC1* and a positive prognosis for overall survival was found in patients with non-small cell lung cancer (75). A functional *MANSC1* Single Nucleotide Polymorphism has been also identified in patients with overall prostate cancer and non-advanced prostate cancer in a genome-wide association study (76). The metabolic enzyme ACP6 hydrolyzes LPA to monoacylglycerol and plays a role in regulating lipid metabolism in the mitochondria (77, 78). It has been recently demonstrated that overexpression of ACP6 in hepatocellular carcinoma tissue was positively correlated with clinical progression and worse overall survival of examined patients (77). On the other hand, decreased expression of ACP6 was found to contribute to increased cell mortality and disease progression in high-grade serous ovarian cancer and esophageal squamous cell carcinoma (78, 79). It was found that CRC cells have abnormal LPA receptor expression that may be associated with enhanced proliferation, survival, and invasion of CRC cells (80). These results suggest that ACP6 may play a key role in oncogenesis. A positive correlation of plasma ACP6 with the advanced stage of CRC has been revealed for the first time in our study. Moreover, ACP6 was reduced in CRC patients with cancer-related inflammation. The function of ACP6 in cancer-related inflammation and CRC tumorigenesis needs to be further investigated.

More interestingly, three pro-inflammatory cytokines, IL32, IL17C, and IFNG, were increased in the early stages of CRC compared to late-stage patients in the Polish cohort. IL32 is an intracellular pluripotent cytokine, expressed in various cell types, which affects many cellular and physiological functions such as cell death and survival, angiogenesis, inflammation, and response to pathogens (81). Increased levels of IL32 were found in cancer tissue (82, 83), and primary CRC lymph nodes metastasis (84). Moreover, IL32 can stimulate NK and T-cell cytotoxicity against primary solid tumors, as well as increase T-cell infiltration (85). In our study, we observed increased circulating IL32 associated with the early tumor stage, indicating that IL32 may serve as a biomarker for the early stage of CRC. The second pro-inflammatory cytokine, IL17C, a member of the IL17 family, plays an essential role in immunopathology, autoimmune diseases, and cancer progression (86). It was found that IL17C is higher expressed in CRC tissue and induces tumor angiogenesis of intestinal endothelial cells *via* VEGFR2 production, subsequently enhancing cell invasion and migration of CRC cells (87, 88). Moreover, elevated levels of serum and tissue IL17C were observed in patients with active IBD, which can result in cancer progression (87). Among these patients, the production of IL17C is induced by the synergic effect of IL17A and TNF-α (89). Therefore, high circulating IL17C may be associated with tumorigenesis from IBD to early stages of CRC. Lasts of these cytokines, IFNG, is critical to both innate and adaptive immunity (90). IFNG was reduced in PBMC of patients with recurrent CRC, with the most significantly reduced expression in stage IV tumors (91). On contrary, the upregulation of *IFNG* mRNA in late-stage CRC tissue and peripheral blood of patients with CRC was observed in another study (92). IFNG is a well-established anti-tumor factor with controversial findings in CRC at mRNA and protein levels. Several studies did not find a significant association between circulating IFNG and CRC development (93–95). In contrast, our analysis showed high levels of IFNG in CRC patients supporting previous findings (31). Moreover, we found high levels of IFNG in the early stages of CRC, suggesting a higher anti-tumor activity of lymphocytes than in the late stages. Taken as a whole, these findings indicate that ACP6, FLT4, MANSC1, IFNG, IL17C, and IL32 may be used as promising prognostic biomarkers that distinguish early-stage from advanced CRC. Moreover, IFNG can be a potential biomarker for early detection of CRC due to its discrimination between early-stage patients with advanced CRC patients as well as healthy controls, which has not been reported before.

In this study, the application of PEA technology enabled us to detect 690 proteins from a low amount of plasma of CRC patients and healthy subjects. Despite the sensitivity and accuracy of PEA, this technology is limited by the availability and specificity of antibodies, and more importantly, the number of preselected proteins. Women are dominant in both study groups, which is different concerning the known population with CRC. We lacked information on family history, which is known as one of the best predictors of CRC risk. Future studies should be conducted to verify our results on a larger number of samples and by using PEA or other quantitative methods.

In conclusion, we identified plasma protein changes in CRC patients related to cytokine interactions, oncogenic pathways, Th17 activity, metabolism reprogramming, as well as cancer-related inflammation with potential usage in CRC diagnosis. We also validated in an independent cohort that ACP6 level was elevated in advanced CRC patients. Further study using larger cohort is needed to confirm whether FLT4, IFNG, IL17C, IL32, and MANSC1 may be used as potential prognostic biomarkers to discriminate early-stage and advanced CRC.

## Data availability statement

The original contributions presented in the study are included in the article/**Supplementary Material**. Further inquiries can be directed to the corresponding author.

## Ethics statement

The study was conducted in accordance with the Declaration of Helsinki, and approved by the Independent Bioethics Committee for Scientific Research at the Medical University of Gdansk (NKBBN/443/2021, 2021-06-15 and NKBBN/564/2018, 2018-11-14). The patients/participants provided their written informed consent to participate in this study.

## Author contributions

Conceptualization, ZC; methodology, VU-S; formal analysis, VU-S; resources, KD-C, NF, AP, JPD, MZ, EŚ, ŁS, MJ, DB, WZ, TN, WM, AAd, AAm, MP, AH-L, JR, and ZC; data curation, AJ, QH, and NF; writing—original draft preparation, AJ; writing—review and editing, DM VU-S, AJ, AP, JD, and ZC; visualization, VU-S; supervision, BL and ZC; project administration, NF, AJ, and ZC; funding acquisition, AP, JPD, and ZC. All authors contributed to the article and approved the submitted version.

## Glossary

**Table:**

ACP6	lysophosphatidic acid phosphatase type 6
AGRP	agouti-related neuropeptidase
APBB1IP	amyloid beta precursor protein binding family B member 1 interacting protein
ARHGEF12	Rho guanine nucleotide exchange factor 12
BCL-2	B-cell lymphoma 2
BID	BH3 interacting domain death agonist
CA11	carbonic anhydrase
CASP8	caspase-8
CCL20	C-C motif chemokine ligand 20
CCR1	C-C Motif Chemokine Receptor 1
CD276	cluster of differentiation 276
CEA	carcinoembryonic antigen
CLEC4G	C-type lectin domain family 4 member G
CRC	Colorectal cancer
CSF3	colony-stimulating factor 3
CXCL6	C-X-C motif chemokine ligand 6
DEPs	differentially expressed proteins
DPEP2	dipeptidase 2
EGFL7	epidermal growth factor-like protein 7
ENPP5	ectonucleotide pyrophosphatase/phosphodiesterase family member 5
FDR	false discovery rate
FGF21	fibroblast growth factor 21
FLT4	Fms-related tyrosine kinase 4
HAGH	hydroxyacylglutathione hydrolase
IBD	inflammatory bowel disease
IFNG	interferon γ
IL	interleukin
IL12RB1	interleukin 12 receptor subunit beta 1
ITGA11	integrin subunit alpha 11
JAK	Janus kinase
KEGG	Kyoto Encyclopedia of Genes and Genomes
LARG	leukemia-associated Rho guanine-nucleotide exchange factor
LOD	limit of detection
LPA	lysophosphatidic acid
MANSC1	MANSC domain-containing protein 1
MAPK	mitogen-activated protein kinase
MDK	midkine
MMP-2/9	matrix metalloproteinase-2/9
MZB1	marginal zone B and B1 cell-specific protein
NAFLD	non-fatty liver disease
NCF2	neutrophil cytosolic factor 2
NF-κB	nuclear factor kappa B
NPX	normalized protein expression
PBMC	peripheral blood mononuclear cells
PEA	proximity extension assay
PPY	pancreatic prohormone
PRDX6	Peroxiredoxin 6
Ras	resistance to audiogenic seizures
RET	Ret proto-oncogen
RRM2B	ribonucleotide reductase regulatory TP53 inducible subunit M2B
RSPO3	R-Spondin 3
S100A12	S100 calcium-binding protein A12
SCGB1A1	secretoglobin family 1A member 1
SCRN1	secernin 1
SELPLG	selectin P ligand
SMOC2	SPARC-related modular calcium-binding protein 2, STAT, signal transducer and activator of transcription
TFF2	trefoil factor 2
TME	tumor microenvironment
TMPRSS15	transmembrane serine protease 15
TNF	tumor necrosis factor
TXNDC15	thioredoxin domain containing 15
TYK2	with tyrosine kinase 2
VEGFR3	Vascular Endothelial Growth Factor Receptor 3
WFIKKN2	WAP, Follilastin/Kazal, Immunoglobulin, Kunitz And Netrin Domain-Containing Protein 2.
