# Supplementary material for: Plasma protein changes reflect colorectal cancer development and associated inflammation
Source: Front Oncol. 2023 May 9;13:1158261. doi: 10.3389/fonc.2023.1158261 (PMC10203952; doi:10.3389/fonc.2023.1158261)
Supplement: Supplementary file 1 [file DataSheet_1.docx]

Supplementary Material

Plasma protein changes reflect colorectal cancer development and associated inflammation

**Víctor Urbiola-Salvador^†^, Agnieszka Jabłońska^†^, Dominika Miroszewska, Qianru Huang, Katarzyna Duzowska, Kinga Drężek-Chyła, Marek Zdrenka, Ewa Śrutek, Łukasz Szylberg, Michał Jankowski, Dariusz Bała, Wojciech Zegarski, Tomasz Nowikiewicz, Wojciech Makarewicz, Agnieszka Adamczyk, Aleksandra Ambicka, Marcin Przewoźnik, Agnieszka Harazin-Lechowicz, Janusz Ryś, Natalia Filipowicz, Arkadiusz Piotrowski, Jan P. Dumanski, Bin Li & Zhi Chen***

*** Correspondence:** Zhi Chen: zhi.chen@ug.edu.pl


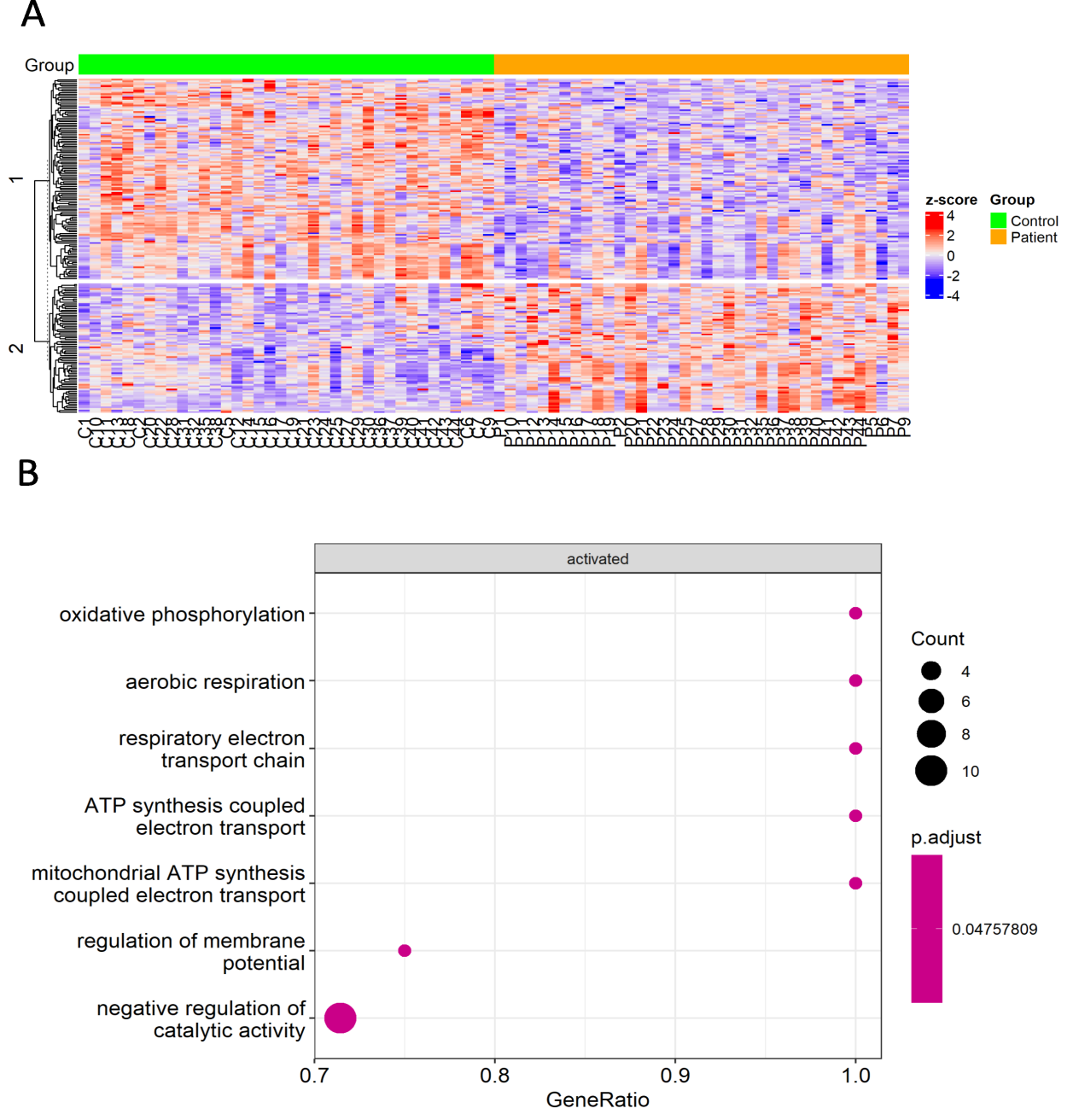


**Supplementary Figure 1.** Plasma protein changes and biological processes induced by colorectal cancer. **(A)** Heatmap of the differentially expressed proteins (DEP) among CRC patients and healthy controls with z-score by row normalization and distributed by hierarchical clustering. The 2 clusters are generated by the K-means algorithm. **(B)** Dot plot with statistically significant Gene Ontology enriched terms from Gene Set Enrichment Analysis (GSEA) after false discovery rate (FDR) correction. p.adjust, adjusted p-value.

**Supplementary Table 1.** List of proteins included in the Olink 384-Oncology Explore panel with their respective Uniprot accession and gene name.

| **Uniprot** | **Gene name** |
| --- | --- |
| Q96SM3 | CPXM1 |
| Q9NZ53 | PODXL2 |
| P12931 | SRC |
| Q02763 | TEK |
| Q92876 | KLK6 |
| O43464 | HTRA2 |
| O95721 | SNAP29 |
| P07237 | P4HB |
| P78552 | IL13RA1 |
| Q9UBX1 | CTSF |
| Q96PD2 | DCBLD2 |
| O43240 | KLK10 |
| P29017 | CD1C |
| P41271 | NBL1 |
| Q9NP84 | TNFRSF12A |
| O00244 | ATOX1 |
| O14974 | PPP1R12A |
| O75629 | CREG1 |
| P06870 | KLK1 |
| P20851 | C4BPB |
| P49767 | VEGFC |
| Q16543 | CDC37 |
| Q5JTD0 | TJAP1 |
| Q6FI81 | CIAPIN1 |
| Q7Z4W1 | DCXR |
| Q8NCC3 | PLA2G15 |
| Q9HAT2 | SIAE |
| Q9UJ68 | MSRA |
| Q9UP79 | ADAMTS8 |
| P20138 | CD33 |
| P21802 | FGFR2 |
| O00548 | DLL1 |
| O43895 | XPNPEP2 |
| O60259 | KLK8 |
| O95274 | LYPD3 |
| P01298 | PPY |
| P04626 | ERBB2 |
| P09486 | SPARC |
| P09958 | FURIN |
| P13688 | CEACAM1 |
| P15328 | FOLR1 |
| P18084 | ITGB5 |
| P21589 | NT5E |
| P21741 | MDK |
| P26447 | S100A4 |
| P26842 | CD27 |
| P35916 | FLT4 |
| P35968 | KDR |
| P37173 | TGFBR2 |
| P41439 | FOLR3 |
| P48307 | TFPI2 |
| Q14508 | WFDC2 |
| Q14512 | FGFBP1 |
| Q15303 | ERBB4 |
| Q16674 | MIA |
| Q96NY8 | NECTIN4 |
| Q9BYH1 | SEZ6L |
| Q9UBX7 | KLK11 |
| P32004 | L1CAM |
| Q92832 | NELL1 |
| Q96J42 | TXNDC15 |
| Q9H8J5 | MANSC1 |
| A4D1B5 | GSAP |
| O00221 | NFKBIE |
| O00451 | GFRA2 |
| O14662 | STX16 |
| O43699 | SIGLEC6 |
| O43752 | STX6 |
| O60243 | HS6ST1 |
| O75054 | IGSF3 |
| O94760 | DDAH1 |
| P01242 | GH2 |
| P01275 | GCG |
| P04637 | TP53 |
| P09758 | TACSTD2 |
| P0CG37 | CFC1 |
| P15848 | ARSB |
| P16562 | CRISP2 |
| P21810 | BGN |
| P23515 | OMG |
| P25685 | DNAJB1 |
| P30041 | PRDX6 |
| P47929 | LGALS7_LGALS7B |
| Q01543 | FLI1 |
| Q02742 | GCNT1 |
| Q07960 | ARHGAP1 |
| Q10471 | GALNT2 |
| Q13561 | DCTN2 |
| Q13576 | IQGAP2 |
| Q16653 | MOG |
| Q49AH0 | CDNF |
| Q6UXD5 | SEZ6L2 |
| Q7Z5A7 | TAFA5 |
| Q7Z5R6 | APBB1IP |
| Q86SJ2 | AMIGO2 |
| Q8N5S9 | CAMKK1 |
| Q8TD06 | AGR3 |
| Q8WYN0 | ATG4A |
| Q92982 | NINJ1 |
| Q96I82 | KAZALD1 |
| Q96PQ0 | SORCS2 |
| Q9C0C4 | SEMA4C |
| Q9H156 | SLITRK2 |
| Q9NX58 | LYAR |
| Q9P0V8 | SLAMF8 |
| Q9UBM4 | OPTC |
| Q9UK85 | DKKL1 |
| Q9UKR0 | KLK12 |
| Q9Y662 | HS3ST3B1 |
| Q9Y6A5 | TACC3 |
| P02760 | AMBP |
| P13726 | F3 |
| P31994 | FCGR2B |
| P35318 | ADM |
| P35475 | IDUA |
| P39900 | MMP12 |
| P47992 | XCL1 |
| Q14213_Q8NEV9 | EBI3_IL27 |
| Q96D42 | HAVCR1 |
| Q99075 | HBEGF |
| Q9BQ51 | PDCD1LG2 |
| Q9UJM8 | HAO1 |
| P01375 | TNF |
| P05231 | IL6 |
| P06127 | CD5 |
| P10145 | CXCL8 |
| P80075 | CCL8 |
| P80511 | S100A12 |
| Q14790 | CASP8 |
| Q8IXJ6 | SIRT2 |
| Q9GZV9 | FGF23 |
| Q9NSA1 | FGF21 |
| O95786 | DDX58 |
| P10747 | CD28 |
| P18627 | LAG3 |
| P34130 | NTF4 |
| P58499 | FAM3B |
| Q05516 | ZBTB16 |
| Q13490 | BIRC2 |
| Q14203 | DCTN1 |
| Q6EIG7 | CLEC6A |
| Q9UQQ2 | SH2B3 |
| O15123 | ANGPT2 |
| O43827 | ANGPTL7 |
| P05089 | ARG1 |
| P26010 | ITGB7 |
| P27695 | APEX1 |
| P51858 | HDGF |
| P98082 | DAB2 |
| Q96JA1 | LRIG1 |
| Q9BZR6 | RTN4R |
| Q9H6B4 | CLMP |
| Q9NPH0 | ACP6 |
| P08473 | MME |
| P28907 | CD38 |
| P29459_P29460 | IL12A_IL12B |
| P56159 | GFRA1 |
| Q16775 | HAGH |
| Q2VWP7 | PRTG |
| Q8TDQ1 | CD300LF |
| Q9HAV5 | EDA2R |
| Q9Y336 | SIGLEC9 |
| O00292 | LEFTY2 |
| O14964 | HGS |
| O15116 | LSM1 |
| O43715 | TRIAP1 |
| O60825 | PFKFB2 |
| O60828 | PQBP1 |
| O75380 | NDUFS6 |
| O75493 | CA11 |
| O95498 | VNN2 |
| P05783 | KRT18 |
| P05937 | CALB1 |
| P10606 | COX5B |
| P14136 | GFAP |
| P22307 | SCP2 |
| P31350 | RRM2 |
| P34949 | MPI |
| P35813 | PPM1A |
| P39748 | FEN1 |
| P41586 | ADCYAP1R1 |
| P43629 | KIR3DL1 |
| P46060 | RANGAP1 |
| P50120 | RBP2 |
| P50583 | NUDT2 |
| P51692 | STAT5B |
| P54727 | RAD23B |
| P55273 | CDKN2D |
| P61218 | POLR2F |
| P82980 | RBP5 |
| Q00796 | SORD |
| Q06787 | FMR1 |
| Q08AG7 | MZT1 |
| Q12846 | STX4 |
| Q15427 | SF3B4 |
| Q16595 | FXN |
| Q16772 | GSTA3 |
| Q3B7J2 | GFOD2 |
| Q6NXT1 | ANKRD54 |
| Q6P2H3 | CEP85 |
| Q6PGN9 | PSRC1 |
| Q6UWN8 | SPINK6 |
| Q6UWW8 | CES3 |
| Q6UX82 | LYPD8 |
| Q7L5N7 | LPCAT2 |
| Q7Z434 | MAVS |
| Q7Z6M1 | RABEPK |
| Q7Z7D3 | VTCN1 |
| Q8IWL1 | SFTPA2 |
| Q8N129 | CNPY4 |
| Q8N386 | LRRC25 |
| Q8N9I9 | DTX3 |
| Q8NBZ7 | UXS1 |
| Q8WUX2 | CHAC2 |
| Q8WWY7 | WFDC12 |
| Q96DU3 | SLAMF6 |
| Q96NA2 | RILP |
| Q96NB1 | CEP20 |
| Q99683 | MAP3K5 |
| Q9BSG5 | RTBDN |
| Q9BSW2 | CRACR2A |
| Q9BUE0 | MED18 |
| Q9C005 | DPY30 |
| Q9H4P4 | RNF41 |
| Q9H6S3 | EPS8L2 |
| Q9HAV7 | GRPEL1 |
| Q9NS15 | LTBP3 |
| Q9NUY8 | TBC1D23 |
| Q9NZT2 | OGFR |
| Q9UQB8 | BAIAP2 |
| Q9Y223 | GNE |
| Q9Y243 | AKT3 |
| Q9Y265 | RUVBL1 |
| Q9Y2Z0 | SUGT1 |
| Q9Y570 | PPME1 |
| Q9Y639 | NPTN |
| O15263 | DEFB4A_DEFB4B |
| P32926 | DSG3 |
| P37108 | SRP14 |
| P40198 | CEACAM3 |
| P43628 | KIR2DL3 |
| Q8IX05 | CD302 |
| Q96EK5 | KIFBP |
| Q9BTE6 | AARSD1 |
| Q9H4A9 | DPEP2 |
| Q9H4F8 | SMOC1 |
| Q9UKS7 | IKZF2 |
| O00186 | STXBP3 |
| O00748 | CES2 |
| O14713 | ITGB1BP1 |
| O15357 | INPPL1 |
| O43570 | CA12 |
| O60760 | HPGDS |
| O75569 | PRKRA |
| O75787 | ATP6AP2 |
| O95831 | AIFM1 |
| P01229 | LHB |
| P06850 | CRH |
| P07332 | FES |
| P07947 | YES1 |
| P09960 | LTA4H |
| P20472 | PVALB |
| P25786 | PSMA1 |
| P35070 | BTC |
| P40121 | CAPG |
| P42658 | DPP6 |
| P49788 | RARRES1 |
| P50749 | RASSF2 |
| P80303 | NUCB2 |
| P98073 | TMPRSS15 |
| Q02246 | CNTN2 |
| Q07954 | LRP1 |
| Q11201 | ST3GAL1 |
| Q13145 | BAMBI |
| Q15116 | PDCD1 |
| Q15797 | SMAD1 |
| Q7L5Y9 | MAEA |
| Q7LG56 | RRM2B |
| Q86SJ6 | DSG4 |
| Q86SR1 | GALNT10 |
| Q86WD7 | SERPINA9 |
| Q8IUK5 | PLXDC1 |
| Q96RT1 | ERBIN |
| Q9GZY6 | LAT2 |
| Q9NRA1 | PDGFC |
| Q9P0J1 | PDP1 |
| Q9UHF1 | EGFL7 |
| Q9ULX7 | CA14 |
| Q9Y5L3 | ENTPD2 |
| Q9Y5V3 | MAGED1 |
| Q9Y653 | ADGRG1 |
| O00592 | PODXL |
| O00622 | CCN1 |
| O14828 | SCAMP3 |
| O60911 | CTSV |
| O75144 | ICOSLG |
| O95388 | CCN4 |
| P00519 | ABL1 |
| P06731 | CEACAM5 |
| P06756 | ITGAV |
| P07948 | LYN |
| P07949 | RET |
| P08069 | IGF1R |
| P15514 | AREG |
| P16870 | CPE |
| P29317 | EPHA2 |
| P35052 | GPC1 |
| P38936 | CDKN1A |
| P50579 | METAP2 |
| Q13421 | MSLN |
| Q16790 | CA9 |
| Q6BAA4 | FCRLB |
| Q8TE58 | ADAMTS15 |
| Q8WXI7 | MUC16 |
| Q99717 | SMAD5 |
| Q9BXY4 | RSPO3 |
| Q9NS68 | TNFRSF19 |
| Q9P0G3 | KLK14 |
| Q9UBG3 | CRNN |
| Q9UJ71 | CD207 |
| Q9UKR3 | KLK13 |
| Q9Y5W5 | WIF1 |
| O00233 | PSMD9 |
| O14558 | HSPB6 |
| O43524 | FOXO3 |
| O60763 | USO1 |
| O60907 | TBL1X |
| O75695 | RP2 |
| P01303 | NPY |
| P05187 | ALPP |
| P08397 | HMBS |
| P09105 | HBQ1 |
| P09110 | ACAA1 |
| P13521 | SCG2 |
| P15121 | AKR1B1 |
| P17948 | FLT1 |
| P30260 | CDC27 |
| P35637 | FUS |
| P36888 | FLT3 |
| P42331 | ARHGAP25 |
| P43490 | NAMPT |
| P48643 | CCT5 |
| P49441 | INPP1 |
| P51580 | TPMT |
| P55008 | AIF1 |
| P55039 | DRG2 |
| P55789 | GFER |
| P62166 | NCS1 |
| Q14241 | ELOA |
| Q496F6 | CD300E |
| Q5VIR6 | VPS53 |
| Q6PCB0 | VWA1 |
| Q7Z5L0 | VMO1 |
| Q86SF2 | GALNT7 |
| Q8IWL2 | SFTPA1 |
| Q8NEZ2 | VPS37A |
| Q96I15 | SCLY |
| Q99536 | VAT1 |
| Q99795 | GPA33 |
| Q9BS26 | ERP44 |
| Q9BSL1 | UBAC1 |
| Q9BYE9 | CDHR2 |
| Q9H3G5 | CPVL |
| Q9NTU7 | CBLN4 |
| Q9P1Z2 | CALCOCO1 |
| Q9UHD8 | SEPTIN9 |
| Q9Y5K2 | KLK4 |
| Q9Y5K8 | ATP6V1D |

**Supplementary Table 2.** List of proteins included in the Olink 384-Inflammation Explore panel with their respective Uniprot accession and gene name.

| **Uniprot** | **Gene name** |
| --- | --- |
| O00182 | LGALS9 |
| O14836 | TNFRSF13B |
| P01127 | PDGFB |
| P18510 | IL1RN |
| P19883 | FST |
| P25116 | F2R |
| P51888 | PRELP |
| Q15109 | AGER |
| Q15389 | ANGPT1 |
| Q16651 | PRSS8 |
| Q8IYS5 | OSCAR |
| Q92583 | CCL17 |
| Q99895 | CTRC |
| O00175 | CCL24 |
| O00626 | CCL22 |
| P16422 | EPCAM |
| P27930 | IL1R2 |
| P36941 | LTBR |
| P56470 | LGALS4 |
| Q03405 | PLAUR |
| Q15166 | PON3 |
| Q92956 | TNFRSF14 |
| Q96PL1 | SCGB3A2 |
| Q9HCB6 | SPON1 |
| Q9NQ76 | MEPE |
| O00241 | SIRPB1 |
| O00339 | MATN2 |
| O00585 | CCL21 |
| O14773 | TPP1 |
| O43291 | SPINT2 |
| O95633 | FSTL3 |
| P15291 | B4GALT1 |
| P19256 | CD58 |
| P24387 | CRHBP |
| P29350 | PTPN6 |
| P47712 | PLA2G4A |
| Q14118 | DAG1 |
| Q16363 | LAMA4 |
| Q4KMG0 | CDON |
| Q5KU26 | COLEC12 |
| Q6GTX8 | LAIR1 |
| Q6UXH1 | CRELD2 |
| Q8TEU8 | WFIKKN2 |
| Q99538 | LGMN |
| Q99983 | OMD |
| Q9BY76 | ANGPTL4 |
| Q9NZV1 | CRIM1 |
| Q9Y6N7 | ROBO1 |
| O00300 | TNFRSF11B |
| O95750 | FGF19 |
| P02778 | CXCL10 |
| P09238 | MMP10 |
| P09341 | CXCL1 |
| P09603 | CSF1 |
| P13236 | CCL4 |
| P25942 | CD40 |
| P55773 | CCL23 |
| Q07325 | CXCL9 |
| Q08334 | IL10RB |
| Q14116 | IL18 |
| Q8NFT8 | DNER |
| P22466 | GAL |
| P46109 | CRKL |
| Q03403 | TFF2 |
| Q6UWV6 | ENPP7 |
| Q9BYZ8 | REG4 |
| Q9UHX3 | ADGRE2 |
| P53634 | CTSC |
| P55145 | MANF |
| Q9BZZ2 | SIGLEC1 |
| Q9H3U7 | SMOC2 |
| O00468 | AGRN |
| O43598 | DNPH1 |
| O43639 | NCK2 |
| O75462 | CRLF1 |
| O75563 | SKAP2 |
| O76096 | CST7 |
| O95866 | MPIG6B |
| P07148 | FABP1 |
| P0DMV8 | HSPA1A |
| P11684 | SCGB1A1 |
| P12532 | CKMT1A_CKMT1B |
| P19876 | CXCL3 |
| P21709 | EPHA1 |
| P29279 | CCN2 |
| P30613 | PKLR |
| P34896 | SHMT1 |
| P35625 | TIMP3 |
| P54317 | PNLIPRP2 |
| Q13232 | NME3 |
| Q14210 | LY6D |
| Q5ZPR3 | CD276 |
| Q7KYR7 | BTN2A1 |
| Q8WU39 | MZB1 |
| Q8WXD2 | SCG3 |
| Q92484 | SMPDL3A |
| Q96KG7 | MEGF10 |
| Q99435 | NELL2 |
| Q99685 | MGLL |
| Q9BU40 | CHRDL1 |
| Q9H008 | LHPP |
| Q9HC38 | GLOD4 |
| Q9NR12 | PDLIM7 |
| Q9NZC2 | TREM2 |
| Q9UII2 | ATP5IF1 |
| Q9UJA9 | ENPP5 |
| Q9UJU6 | DBNL |
| Q9UKU9 | ANGPTL2 |
| Q9Y3D6 | FIS1 |
| O75888 | TNFSF13 |
| P01133 | EGF |
| P09326 | CD48 |
| P15260 | IFNGR1 |
| P21860 | ERBB3 |
| Q9NQ30 | ESM1 |
| O14904 | WNT9A |
| O43521-2 | BCL2L11 |
| O95644 | NFATC1 |
| P11274 | BCR |
| P52564 | MAP2K6 |
| P57771 | RGS8 |
| P58294 | PROK1 |
| Q06520 | SULT2A1 |
| Q6UB28 | METAP1D |
| Q6UXK5 | LRRN1 |
| Q969V3 | NCLN |
| Q96LC7 | SIGLEC10 |
| Q9HCU5 | PREB |
| Q9NRM6 | IL17RB |
| Q9NZN5 | ARHGEF12 |
| O00253 | AGRP |
| O43915 | VEGFD |
| P01730 | CD4 |
| P09874 | PARP1 |
| P24394 | IL4R |
| P26022 | PTX3 |
| P29965 | CD40LG |
| P49763 | PGF |
| Q12866 | MERTK |
| Q13219 | PAPPA |
| Q14005 | IL16 |
| Q14242 | SELPLG |
| Q16698 | DECR1 |
| Q8TAD2 | IL17D |
| Q9HB29 | IL1RL2 |
| Q9NQ25 | SLAMF7 |
| Q9UIB8 | CD84 |
| Q9Y6K9 | IKBKG |
| Q9Y6Q6 | TNFRSF11A |
| O14788 | TNFSF11 |
| O15169 | AXIN1 |
| O15444 | CCL25 |
| O43508 | TNFSF12 |
| O95760 | IL33 |
| P00813 | ADA |
| P01135 | TGFA |
| P01137 | TGFB1 |
| P01374 | LTA |
| P01375 | TNF |
| P01579 | IFNG |
| P01583 | IL1A |
| P03956 | MMP1 |
| P05112 | IL4 |
| P05113 | IL5 |
| P05231 | IL6 |
| P10145 | CXCL8 |
| P10147 | CCL3 |
| P12034 | FGF5 |
| P13232 | IL7 |
| P13725 | OSM |
| P14210 | HGF |
| P14784 | IL2RB |
| P15692 | VEGFA |
| P20783 | NTF3 |
| P22301 | IL10 |
| P29460 | IL12B |
| P30203 | CD6 |
| P35225 | IL13 |
| P42702 | LIFR |
| P49771 | FLT3LG |
| P50591 | TNFSF10 |
| P51671 | CCL11 |
| P60568 | IL2 |
| P78556 | CCL20 |
| P80098 | CCL7 |
| P80162 | CXCL6 |
| Q13007 | IL24 |
| Q13261 | IL15RA |
| Q13291 | SLAMF1 |
| Q13478 | IL18R1 |
| Q13651 | IL10RA |
| Q16552 | IL17A |
| Q5T4W7 | ARTN |
| Q8N6P7 | IL22RA1 |
| Q99616 | CCL13 |
| Q99748 | NRTN |
| Q9BZW8 | CD244 |
| Q9NRJ3 | CCL28 |
| Q9NYY1 | IL20 |
| Q9P0M4 | IL17C |
| Q9UHF4 | IL20RA |
| O00273 | DFFA |
| O14867 | BACH1 |
| O43597 | SPRY2 |
| O43736 | ITM2A |
| O60449 | LY75 |
| O60880 | SH2D1A |
| O75475 | PSIP1 |
| O76036 | NCR1 |
| O94992 | HEXIM1 |
| P05412 | JUN |
| P08727 | KRT19 |
| P09038 | FGF2 |
| P14317 | HCLS1 |
| P16455 | MGMT |
| P18564 | ITGB6 |
| P19474 | TRIM21 |
| P23229 | ITGA6 |
| P27540 | ARNT |
| P28845 | HSD11B1 |
| P30044 | PRDX5 |
| P30048 | PRDX3 |
| P42701 | IL12RB1 |
| P48061 | CXCL12 |
| P51617 | IRAK1 |
| P63241 | EIF5A |
| P78310 | CXADR |
| P78362 | SRPK2 |
| P78410 | BTN3A2 |
| Q01151 | CD83 |
| Q03431 | PTH1R |
| Q04637 | EIF4G1 |
| Q04759 | PRKCQ |
| Q05084 | ICA1 |
| Q07065 | CKAP4 |
| Q12933 | TRAF2 |
| Q12968 | NFATC3 |
| Q13241 | KLRD1 |
| Q13574 | DGKZ |
| Q14435 | GALNT3 |
| Q15517 | CDSN |
| Q15661 | TPSAB1 |
| Q6DN72 | FCRL6 |
| Q6UXB4 | CLEC4G |
| Q6ZUJ8 | PIK3AP1 |
| Q7Z6M3 | MILR1 |
| Q8IU57 | IFNLR1 |
| Q8N608 | DPP10 |
| Q8NHJ6 | LILRB4 |
| Q8WTT0 | CLEC4C |
| Q8WXI8 | CLEC4D |
| Q92844 | TANK |
| Q96DB9 | FXYD5 |
| Q96P31 | FCRL3 |
| Q96SB3 | PPP1R9B |
| Q9BXN2 | CLEC7A |
| Q9C035 | TRIM5 |
| Q9GZT9 | EGLN1 |
| Q9HCM2 | PLXNA4 |
| Q9NWZ3 | IRAK4 |
| Q9UHC6 | CNTNAP2 |
| Q9UKX5 | ITGA11 |
| Q9UMR7 | CLEC4A |
| Q9UN19 | DAPP1 |
| Q9UNE0 | EDAR |
| Q9UQV4 | LAMP3 |
| Q9Y2J8 | PADI2 |
| Q9Y3P8 | SIT1 |
| P40259 | CD79B |
| P43234 | CTSO |
| P50452 | SERPINB8 |
| P50995 | ANXA11 |
| Q9H4D0 | CLSTN2 |
| O43561 | LAT |
| O75077 | ADAM23 |
| P09919 | CSF3 |
| P12544 | GZMA |
| P41217 | CD200 |
| Q01344 | IL5RA |
| Q16719 | KYNU |
| Q8TD46 | CD200R1 |
| Q96LA5 | FCRL2 |
| B1AKI9 | ISM1 |
| O60884 | DNAJA2 |
| O94856 | NFASC |
| P01903 | HLA-DRA |
| P20809 | IL11 |
| P22304 | IDS |
| P35613 | BSG |
| P36959 | GMPR |
| P37235 | HPCAL1 |
| P45984 | MAPK9 |
| Q12765 | SCRN1 |
| Q12918 | KLRB1 |
| Q3KPI0 | CEACAM21 |
| Q5R372 | RABGAP1L |
| Q6ZMH5 | SLC39A5 |
| Q8IVG5 | SAMD9L |
| Q8TCS8 | PNPT1 |
| Q8WV07 | LTO1 |
| Q92609 | TBC1D5 |
| Q96AX2 | RAB37 |
| Q9BT73 | PSMG3 |
| Q9NP70 | AMBN |
| Q9UPV0 | CEP164 |
| Q9Y258 | CCL26 |
| Q9Y266 | NUDC |
| P19801 | AOC1 |
| P24001 | IL32 |
| P24071 | FCAR |
| P26951 | IL3RA |
| P32456 | GBP2 |
| P40933 | IL15 |
| Q96RJ3 | TNFRSF13C |
| O60934 | NBN |
| P01588 | EPO |
| P19878 | NCF2 |
| P23582 | NPPC |
| P30838 | ALDH3A1 |
| P42768 | WAS |
| P55957 | BID |
| P68106 | FKBP1B |
| Q03426 | MVK |
| Q0Z7S8 | FABP9 |
| Q12778 | FOXO1 |
| Q7L8A9 | VASH1 |
| Q8N8S7 | ENAH |
| Q8NDB2 | BANK1 |
| Q9BXJ7 | AMN |
| Q9UNK0 | STX8 |
| Q9Y478 | PRKAB1 |
| Q9Y5A7 | NUB1 |
| O15455 | TLR3 |
| O95971 | CD160 |
| P10144 | GZMB |
| P32970 | CD70 |
| P43489 | TNFRSF4 |
| P48023 | FASLG |
| Q29980_Q29983 | MICB_MICA |
| Q6UXB2 | CXCL17 |
| Q9HBG7 | LY9 |
| O43707 | ACTN4 |
| O60542 | PSPN |
| O60575 | SPINK4 |
| O76038 | SCGN |
| O95379 | TNFAIP8 |
| O95715 | CXCL14 |
| P01584 | IL1B |
| P01591 | JCHAIN |
| P02745 | C1QA |
| P12872 | MLN |
| P13693 | TPT1 |
| P13747 | HLA-E |
| P20273 | CD22 |
| P20340 | RAB6A |
| P20849 | COL9A1 |
| P28827 | PTPRM |
| P28838 | LAP3 |
| P33241 | LSP1 |
| P42575 | CASP2 |
| Q08174 | PCDH1 |
| Q13459 | MYO9B |
| Q14773 | ICAM4 |
| Q7Z739 | YTHDF3 |
| Q96PD4 | IL17F |
| Q9H0P0 | NT5C3A |
| Q9HD26 | GOPC |
| Q9UDT6 | CLIP2 |

**Supplementary Table 3.** DEPs between patients and age and sex-matched healthy controls. The Fold Change is defined as Patient-Healthy control (P-C).

| Protein | logFC(P-C) | adj.p.value |
| --- | --- | --- |
| DPEP2 | 0.927 | 3.56E-16 |
| NCF2 | -1.929 | 4.84E-15 |
| EGFL7 | -0.824 | 1.64E-10 |
| HAGH | 3.090 | 2.59E-10 |
| ENPP5 | -0.852 | 1.13E-09 |
| AGRP | 1.454 | 2.28E-09 |
| MAPK9 | 1.108 | 1.19E-08 |
| ANGPTL4 | 0.892 | 2.14E-08 |
| PRDX6 | 1.470 | 3.47E-08 |
| BID | 2.151 | 5.79E-08 |
| MDK | 1.554 | 5.85E-08 |
| ANGPTL7 | -0.674 | 6.44E-07 |
| INPP1 | 1.260 | 6.44E-07 |
| NDUFS6 | 1.636 | 1.1E-06 |
| COX5B | 1.466 | 2.45E-06 |
| DSG3 | -0.573 | 3.52E-06 |
| CKMT1A_CKMT1B | -1.556 | 4.7E-06 |
| KLK13 | -1.100 | 5.06E-06 |
| AREG | 0.882 | 5.26E-06 |
| LAP3 | 1.075 | 5.65E-06 |
| CRACR2A | -1.898 | 6.49E-06 |
| ITGB1BP1 | 0.776 | 6.49E-06 |
| PODXL | -0.283 | 7.23E-06 |
| ERBB4 | -0.388 | 7.64E-06 |
| CD58 | -0.333 | 1.01E-05 |
| PLXNA4 | -1.516 | 1.29E-05 |
| MAP3K5 | -1.682 | 1.73E-05 |
| CLEC4A | -0.538 | 1.73E-05 |
| ESM1 | 1.056 | 2E-05 |
| ADAMTS8 | -0.614 | 2.4E-05 |
| MPI | 1.767 | 2.71E-05 |
| CASP8 | 1.537 | 2.91E-05 |
| CRH | -1.144 | 4.01E-05 |
| CCL23 | 0.547 | 4.01E-05 |
| GZMB | -0.940 | 4.48E-05 |
| LGALS7_LGALS7B | 0.664 | 5.39E-05 |
| PFKFB2 | -1.357 | 6.69E-05 |
| POLR2F | 0.483 | 8.87E-05 |
| TBC1D5 | -1.177 | 8.92E-05 |
| CD1C | -0.325 | 0.000154 |
| DNER | -0.346 | 0.000164 |
| FLT3 | -0.539 | 0.000175 |
| RET | -0.716 | 0.000183 |
| TAFA5 | 0.509 | 0.000209 |
| TPMT | 1.547 | 0.000215 |
| SPARC | -1.098 | 0.00026 |
| ATP5IF1 | 1.803 | 0.000285 |
| AMIGO2 | -0.307 | 0.00031 |
| PLA2G15 | -0.297 | 0.000366 |
| UXS1 | -0.606 | 0.000436 |
| ADGRE2 | -0.382 | 0.000468 |
| ITGB6 | -0.370 | 0.000532 |
| LSM1 | 0.391 | 0.000538 |
| CTRC | -0.975 | 0.000538 |
| SMOC1 | 0.647 | 0.000624 |
| LRRN1 | -0.480 | 0.000751 |
| RNF41 | -1.025 | 0.000761 |
| TMPRSS15 | -1.132 | 0.000788 |
| RRM2B | -0.831 | 0.000788 |
| PTX3 | 0.681 | 0.000788 |
| CD40LG | -1.146 | 0.000846 |
| MYO9B | -0.460 | 0.000883 |
| SKAP2 | -1.507 | 0.000931 |
| NME3 | -0.307 | 0.000931 |
| IL1R2 | -0.250 | 0.000931 |
| ST3GAL1 | -0.372 | 0.001318 |
| MZB1 | 0.565 | 0.001318 |
| CD84 | -0.507 | 0.001591 |
| FKBP1B | 1.631 | 0.001598 |
| MPIG6B | -1.278 | 0.00163 |
| ADM | 0.736 | 0.00163 |
| RASSF2 | -0.610 | 0.001637 |
| FCGR2B | -0.462 | 0.001698 |
| LTA | -0.380 | 0.001698 |
| CPXM1 | -0.783 | 0.001905 |
| SCRN1 | 0.804 | 0.001981 |
| FLT1 | -0.269 | 0.001982 |
| DNAJA2 | -1.233 | 0.002087 |
| ARHGEF12 | -1.246 | 0.002277 |
| DPP6 | -0.365 | 0.002424 |
| GFRA1 | 0.375 | 0.002643 |
| PDGFB | -1.022 | 0.002732 |
| MERTK | -0.268 | 0.002732 |
| KYNU | -0.701 | 0.002781 |
| LPCAT2 | -0.474 | 0.003133 |
| FGF21 | 1.102 | 0.003148 |
| MAP2K6 | -1.112 | 0.003305 |
| LRP1 | -0.351 | 0.003522 |
| EGF | -1.403 | 0.003585 |
| ICOSLG | -0.196 | 0.003806 |
| CSF1 | 0.384 | 0.003806 |
| TNFSF10 | -0.371 | 0.003866 |
| GALNT7 | -0.248 | 0.003902 |
| CBLN4 | -0.427 | 0.004005 |
| FASLG | -0.392 | 0.004078 |
| MEGF10 | -0.351 | 0.004706 |
| IDS | -0.174 | 0.004917 |
| VEGFC | -0.568 | 0.005002 |
| CREG1 | -0.342 | 0.005048 |
| DPY30 | 0.632 | 0.005179 |
| PRTG | -0.253 | 0.00519 |
| PRKCQ | -0.152 | 0.00562 |
| S100A12 | -1.628 | 0.005715 |
| CALCOCO1 | -1.231 | 0.005715 |
| LYPD3 | -0.310 | 0.005715 |
| CD200 | -0.223 | 0.005715 |
| NELL1 | -0.432 | 0.005799 |
| ICAM4 | -0.432 | 0.006208 |
| TNFRSF13C | -0.390 | 0.006393 |
| STX4 | -0.823 | 0.006512 |
| LY75 | -0.332 | 0.006738 |
| FGFBP1 | 0.642 | 0.006801 |
| LSP1 | -0.531 | 0.006959 |
| IFNG | 0.926 | 0.007133 |
| CSF3 | 0.479 | 0.007316 |
| CCL20 | 0.926 | 0.007485 |
| HBEGF | -0.758 | 0.008658 |
| FST | 0.425 | 0.008658 |
| F2R | 0.955 | 0.008658 |
| TRIAP1 | 0.753 | 0.008765 |
| OMG | -0.536 | 0.008776 |
| GALNT2 | -0.297 | 0.0089 |
| CFC1 | -0.447 | 0.008955 |
| DSG4 | -0.330 | 0.008955 |
| SEZ6L | -0.236 | 0.008955 |
| PREB | 0.259 | 0.008955 |
| RSPO3 | 0.316 | 0.008955 |
| EDAR | -0.906 | 0.009737 |
| PON3 | -0.380 | 0.009795 |
| SLAMF8 | 0.432 | 0.010717 |
| WNT9A | 0.389 | 0.010897 |
| HPGDS | -0.313 | 0.010973 |
| KRT18 | 0.745 | 0.011047 |
| HS3ST3B1 | 0.301 | 0.01114 |
| SLC39A5 | 0.378 | 0.011342 |
| SEZ6L2 | -0.237 | 0.011536 |
| PCDH1 | -0.150 | 0.011571 |
| VAT1 | 0.206 | 0.012038 |
| PPME1 | 0.947 | 0.012038 |
| FGF23 | 0.680 | 0.012041 |
| L1CAM | -0.233 | 0.012302 |
| CD300E | 0.470 | 0.012981 |
| TRIM21 | 0.774 | 0.013175 |
| CXCL9 | 0.543 | 0.01346 |
| SEMA4C | -0.225 | 0.014324 |
| TNFSF12 | -0.364 | 0.0144 |
| CPVL | -0.332 | 0.0144 |
| TPP1 | -0.319 | 0.0144 |
| RARRES1 | -0.196 | 0.0144 |
| IL17A | -0.339 | 0.01544 |
| SPON1 | 0.340 | 0.015855 |
| CTSV | -0.370 | 0.015937 |
| LGALS4 | 0.493 | 0.016288 |
| MILR1 | -0.348 | 0.016899 |
| SCAMP3 | -0.857 | 0.017433 |
| CRNN | -0.544 | 0.017433 |
| IL6 | 0.803 | 0.019437 |
| TPSAB1 | -0.559 | 0.020231 |
| CD22 | -0.408 | 0.020231 |
| GALNT10 | -0.217 | 0.020899 |
| MLN | 0.890 | 0.020899 |
| MAGED1 | 0.164 | 0.020928 |
| NCS1 | -0.281 | 0.02119 |
| MMP12 | 0.575 | 0.021233 |
| SFTPA2 | -0.389 | 0.023031 |
| EPCAM | -0.638 | 0.023229 |
| IL16 | -0.355 | 0.023275 |
| NUDT2 | 0.538 | 0.023275 |
| GH2 | -0.873 | 0.024329 |
| DAB2 | 0.918 | 0.027401 |
| APEX1 | -0.649 | 0.027919 |
| ARSB | -0.641 | 0.028904 |
| BIRC2 | -0.410 | 0.029445 |
| DAG1 | -0.424 | 0.029635 |
| VPS37A | 0.657 | 0.029635 |
| SORD | 0.833 | 0.029635 |
| COL9A1 | -0.379 | 0.029945 |
| SIGLEC6 | -0.247 | 0.031689 |
| IL15 | 0.202 | 0.031689 |
| CKAP4 | 0.238 | 0.031689 |
| GPA33 | -1.037 | 0.032636 |
| CXCL8 | 0.637 | 0.033072 |
| CD28 | -0.286 | 0.033192 |
| NUCB2 | 0.303 | 0.033192 |
| TNFSF11 | -0.547 | 0.033496 |
| IGSF3 | -0.244 | 0.033665 |
| SPINK4 | 0.652 | 0.033665 |
| EDA2R | 0.429 | 0.034567 |
| MMP1 | -0.758 | 0.03477 |
| NPTN | -0.283 | 0.03553 |
| NELL2 | -0.237 | 0.036585 |
| NPPC | 0.338 | 0.037555 |
| LGALS9 | 0.258 | 0.038647 |
| TBC1D23 | -0.555 | 0.039635 |
| SMPDL3A | -0.431 | 0.041561 |
| VEGFD | -0.310 | 0.041561 |
| CXADR | 0.293 | 0.042035 |
| HGF | 0.379 | 0.04454 |
| UBAC1 | 0.414 | 0.04454 |
| LYN | 0.710 | 0.048361 |
| IL12RB1 | 0.237 | 0.049529 |
| STXBP3 | 0.294 | 0.049529 |

**Supplementary Table 4.** KEGG enriched terms for the 202 DEPs between CRC patients and healthy controls. Each term contains an associated description, the size of the gene set, the enrichment score, the normal enrichment score (NES), the p-value; adjusted p-value, q-value, the rank, and the core enrichment with the ENTREZ identifiers of the enriched proteins.

| ID | Term_Description | Fold_Enrichment | occurrence | support | lowest_p-value | highest_p-value | Up_regulated | Down_regulated |
| --- | --- | --- | --- | --- | --- | --- | --- | --- |
| hsa04060 | Cytokine-cytokine receptor interaction | 58 | 10 | 0.2414511 | 2.6e-17 | 3.1e-15 | CCL23, CCL20, CXCL8, CXCL9, IL15, CSF3, IL6, IFNG, CSF1, IL12RB1, EDA2R | GH2, IL16, LTA, FASLG, TNFSF10, TNFSF11, TNFSF12, CD40LG, IL1R2, EDAR, TNFRSF13C |
| hsa04668 | TNF signaling pathway | 82 | 10 | 0.3859649 | 2.2e-14 | 9.0e-10 | MAPK9, CASP8, CCL20, CSF1, IL6, IL15 | BIRC2, MAP2K6, MAP3K5, LTA, VEGFC, VEGFD |
| hsa05323 | Rheumatoid arthritis | 88 | 10 | 0.2586207 | 3.3e-12 | 2.1e-11 | IL15, IL6, CSF1, CCL20, CXCL8, IFNG | CD28, TNFSF11, MMP1, FLT1 |
| hsa05144 | Malaria | 108 | 10 | 0.3419053 | 5.6e-12 | 2.5e-09 | HGF, CXCL8, IL6, CSF3, IFNG | LRP1, CD40LG |
| hsa04061 | Viral protein interaction with cytokine and cytokine receptor | 62 | 10 | 0.1896552 | 4.3e-11 | 5.1e-08 | CCL23, CCL20, CXCL8, CXCL9, IL6, CSF1 | LTA, TNFSF10 |
| hsa04010 | MAPK signaling pathway | 42 | 10 | 0.3304598 | 1.5e-10 | 4.2e-09 | AREG, FGF21, FGF23, CSF1, HGF, MAPK9 | EGF, PDGFB, VEGFC, VEGFD, ERBB4, FLT3, FLT1, FASLG, MAP3K5, MAP2K6 |
| hsa04014 | Ras signaling pathway | 41 | 10 | 0.2956745 | 3.4e-10 | 3.1e-09 | FGF21, FGF23, CSF1, HGF, MAPK9 | EGF, PDGFB, VEGFC, VEGFD, FLT3, FLT1, FASLG |
| hsa04015 | Rap1 signaling pathway | 41 | 10 | 0.2608893 | 3.4e-10 | 1.7e-06 | F2R, FGF21, FGF23, CSF1, HGF | EGF, PDGFB, VEGFC, VEGFD, FLT1, MAP2K6 |
| hsa05417 | Lipid and atherosclerosis | 46 | 10 | 0.3878977 | 4.2e-10 | 5.1e-10 | MAPK9, CXCL8, IL6, LYN, CASP8, BID | MMP1, CD40LG, NCF2, MAP2K6, MAP3K5, TNFSF10, FASLG |
| hsa04064 | NF-kappa B signaling pathway | 76 | 10 | 0.2735243 | 8.5e-10 | 1.4e-07 | LYN, EDA2R, CXCL8 | PRKCQ, BIRC2, EDAR, CD40LG, TNFSF11, LTA, TNFRSF13C |
| hsa04630 | JAK-STAT signaling pathway | 38 | 10 | 0.2749375 | 9.1e-10 | 7.6e-05 | IL6, IL15, IFNG, CSF3, IL12RB1 | GH2, EGF, PDGFB |
| hsa04210 | Apoptosis | 52 | 10 | 0.2413793 | 1.5e-09 | 1.6e-09 | CASP8, BID, MAPK9 | TNFSF10, FASLG, GZMB, CTSV, BIRC2, MAP3K5 |
| hsa04657 | IL-17 signaling pathway | 65 | 10 | 0.3674752 | 2.0e-09 | 3.5e-09 | CASP8, MAPK9, CXCL8, CCL20, IL6, CSF3, IFNG | MMP1 |
| hsa04510 | Focal adhesion | 39 | 10 | 0.2631579 | 2.9e-09 | 2.2e-06 | HGF, MAPK9 | COL9A1, ITGB6, PDGFB, EGF, VEGFC, VEGFD, FLT1, BIRC2 |
| hsa05142 | Chagas disease | 46 | 10 | 0.3275862 | 3.5e-09 | 2.5e-07 | MAPK9, IL6, CXCL8, IFNG, CASP8 | FASLG |
| hsa04659 | Th17 cell differentiation | 36 | 10 | 0.1770050 | 3.5e-09 | 2.5e-07 | IL6, IL12RB1, MAPK9, IFNG | PRKCQ |
| hsa05218 | Melanoma | 54 | 10 | 0.2434967 | 5.6e-09 | 2.0e-08 | FGF21, FGF23, HGF | PDGFB, EGF |
| hsa04020 | Calcium signaling pathway | 36 | 10 | 0.2105263 | 1.0e-08 | 1.7e-06 | F2R, FGF21, FGF23, HGF | EGF, PDGFB, VEGFC, VEGFD, ERBB4, FLT1, RET |
| hsa04012 | ErbB signaling pathway | 49 | 10 | 0.1724138 | 3.3e-08 | 5.1e-08 | AREG, MAPK9 | EGF, HBEGF, ERBB4 |
| hsa04672 | Intestinal immune network for IgA production | 101 | 10 | 0.1217483 | 3.7e-08 | 1.2e-07 | IL6, IL15 | CD28, TNFRSF13C, CD40LG, ICOSLG |
| hsa05164 | Influenza A | 37 | 10 | 0.3077148 | 7.5e-08 | 1.3e-06 | IL6, CXCL8, IFNG, CASP8, BID | TPSAB1, TNFSF10, FASLG |
| hsa05167 | Kaposi sarcoma-associated herpesvirus infection | 32 | 10 | 0.3826376 | 8.1e-08 | 1.9e-06 | IL6, CASP8, BID, MAPK9, LYN, CXCL8 | MAP2K6, PDGFB |
| hsa04620 | Toll-like receptor signaling pathway | 45 | 10 | 0.3596059 | 1.3e-07 | 2.3e-07 | CASP8, MAPK9, IL6, CXCL8, CXCL9 | MAP2K6 |
| hsa00512 | Mucin type O-glycan biosynthesis | 89 | 10 | 0.0172414 | 1.5e-07 | 1.5e-07 |  | GALNT10, GALNT2, GALNT7, ST3GAL1 |
| hsa04658 | Th1 and Th2 cell differentiation | 34 | 10 | 0.1239035 | 1.7e-07 | 6.2e-06 | IFNG, IL12RB1, MAPK9 | PRKCQ |
| hsa05145 | Toxoplasmosis | 42 | 10 | 0.2956745 | 2.0e-07 | 2.8e-07 | IFNG, MAPK9, CASP8 | MAP2K6, BIRC2, CD40LG |
| hsa04660 | T cell receptor signaling pathway | 39 | 10 | 0.2456897 | 2.0e-07 | 1.5e-06 | MAPK9, IFNG | PRKCQ, CD28, CD40LG |
| hsa00240 | Pyrimidine metabolism | 43 | 9 | 0.0172414 | 3.1e-07 | 3.1e-07 | NUDT2 | NME3, RRM2B |
| hsa04217 | Necroptosis | 34 | 10 | 0.2124060 | 3.6e-07 | 1.3e-06 | CASP8, MAPK9, IFNG, BID | BIRC2, TNFSF10, FASLG |
| hsa04810 | Regulation of actin cytoskeleton | 25 | 10 | 0.1754386 | 4.9e-07 | 1.7e-06 | F2R, FGF21, FGF23 | EGF, PDGFB, ITGB6, ARHGEF12 |
| hsa04932 | Non-alcoholic fatty liver disease | 46 | 10 | 0.3613624 | 6.5e-07 | 1.0e-06 | IL6, MAPK9, CXCL8, CASP8, BID, NDUFS6, COX5B | MAP3K5, FASLG |
| hsa05132 | Salmonella infection | 25 | 10 | 0.2772753 | 7.2e-07 | 3.0e-06 | MAPK9, IL6, CXCL8, CASP8 | PODXL, MAP2K6, BIRC2, TNFSF10 |
| hsa05161 | Hepatitis B | 34 | 10 | 0.3445449 | 7.3e-07 | 1.3e-06 | MAPK9, CASP8, BID, CXCL8, IL6 | FASLG, MAP2K6 |
| hsa04062 | Chemokine signaling pathway | 20 | 10 | 0.0938232 | 7.9e-07 | 1.5e-04 | CXCL8, CXCL9, CCL23, CCL20, LYN | |
| hsa04215 | Apoptosis - multiple species | 98 | 10 | 0.1391410 | 9.0e-07 | 1.1e-06 | CASP8, BID, MAPK9 | BIRC2 |
| hsa05134 | Legionellosis | 40 | 10 | 0.2068966 | 1.0e-06 | 1.7e-06 | CASP8, IL6, CXCL8 |  |
| hsa04621 | NOD-like receptor signaling pathway | 21 | 10 | 0.2185028 | 1.1e-06 | 2.2e-06 | IL6, CXCL8, MAPK9, CASP8 | BIRC2 |
| hsa05130 | Pathogenic Escherichia coli infection | 34 | 10 | 0.3135057 | 1.1e-06 | 2.2e-06 | F2R, MAPK9, IL6, CXCL8, CASP8 | ARHGEF12, TNFSF10, FASLG |
| hsa05330 | Allograft rejection | 11 | 10 | 0.1217483 | 1.4e-06 | 1.6e-06 | IFNG | CD28, FASLG, GZMB, CD40LG |
| hsa05152 | Tuberculosis | 35 | 10 | 0.2608893 | 1.5e-06 | 4.9e-05 | CASP8, BID, IFNG, MAPK9, IL6 | ARHGEF12, LSP1, FCGR2B |
| hsa05321 | Inflammatory bowel disease | 37 | 10 | 0.0869631 | 1.5e-06 | 1.0e-04 | IFNG, IL12RB1, IL6 |  |
| hsa04622 | RIG-I-like receptor signaling pathway | 33 | 10 | 0.1367621 | 1.5e-06 | 2.7e-06 | CASP8, MAPK9,  CXCL8 | |
| hsa05169 | Epstein-Barr virus infection | 27 | 10 | 0.2436405 | 1.5e-06 | 2.3e-05 | IL6, CASP8, BID, MAPK9, LYN | MAP2K6, CD58 |
| hsa05120 | Epithelial cell signaling in Helicobacter pylori infection | 43 | 10 | 0.1551724 | 1.6e-06 | 5.7e-06 | CXCL8, LYN, MAPK9 | HBEGF |
| hsa05332 | Graft-versus-host disease | 100 | 10 | 0.1896552 | 1.8e-06 | 1.8e-06 | IL6, IFNG | CD28, FASLG, GZMB |
| hsa04650 | Natural killer cell mediated cytotoxicity | 33 | 10 | 0.1034483 | 1.9e-06 | 6.2e-04 | IFNG, BID | TNFSF10, FASLG, GZMB |
| hsa05133 | Pertussis | 41 | 10 | 0.2413793 | 1.9e-06 | 3.1e-06 | MAPK9, IL6, CXCL8 | SFTPA2 |
| hsa05146 | Amoebiasis | 39 | 10 | 0.2241379 | 2.1e-06 | 5.6e-05 | IL6, CXCL8, IFNG | IL1R2, CD1C |
| hsa04722 | Neurotrophin signaling pathway | 26 | 10 | 0.1825061 | 3.0e-06 | 2.7e-05 | MAPK9, MAGED1 | FASLG, MAP3K5 |
| hsa04115 | p53 signaling pathway | 32 | 10 | 0.0689655 | 3.1e-06 | 1.0e-04 | CASP8, BID | RRM2B |
| hsa05162 | Measles | 39 | 10 | 0.3275862 | 3.6e-06 | 2.0e-05 | MAPK9, IL6, CASP8, BID | FASLG, CD28, FCGR2B |
| hsa04933 | AGE-RAGE signaling pathway in diabetic complications | 39 | 10 | 0.3103448 | 4.7e-06 | 7.8e-06 | IL6, CXCL8, MAPK9 | VEGFC, VEGFD |
| hsa00230 | Purine metabolism | 18 | 9 | 0.0172414 | 4.8e-06 | 4.8e-06 | NUDT2 | NME3, RRM2B |
| hsa04940 | Type I diabetes mellitus | 92 | 10 | 0.0695705 | 4.8e-06 | 1.0e-04 | IFNG | CD28, FASLG, GZMB, LTA |
| hsa04625 | C-type lectin receptor signaling pathway | 37 | 10 | 0.2434967 | 5.1e-06 | 8.4e-06 | CASP8, IL6, MAPK9 | LSP1, ARHGEF12 |
| hsa05171 | Coronavirus disease - COVID-19 | 21 | 10 | 0.1565336 | 5.2e-06 | 7.6e-06 | IL6, CXCL8, CSF3, MAPK9 | MMP1, HBEGF |
| hsa04931 | Insulin resistance | 21 | 10 | 0.1913188 | 5.9e-06 | 9.7e-06 | IL6, MAPK9 | PRKCQ |
| hsa05163 | Human cytomegalovirus infection | 25 | 10 | 0.2735243 | 6.5e-06 | 1.1e-04 | CXCL8, IL6, CASP8, BID | ARHGEF12, MAP2K6, FASLG |
| hsa05224 | Breast cancer | 27 | 10 | 0.1206897 | 7.3e-06 | 1.7e-05 | FGF21, FGF23, WNT9A | TNFSF11, EGF |
| hsa04068 | FoxO signaling pathway | 30 | 10 | 0.2586207 | 1.0e-05 | 1.0e-05 | MAPK9, IL6 | EGF, FASLG, TNFSF10 |
| hsa05170 | Human immunodeficiency virus 1 infection | 18 | 10 | 0.2137002 | 1.1e-05 | 2.7e-05 | MAPK9, CASP8, BID | MAP2K6, FASLG |
| hsa05235 | PD-L1 expression and PD-1 checkpoint pathway in cancer | 43 | 10 | 0.2456897 | 1.2e-05 | 4.4e-05 | IFNG | EGF, MAP2K6, CD28, PRKCQ |
| hsa05135 | Yersinia infection | 34 | 10 | 0.2758621 | 1.3e-05 | 2.1e-05 | MAPK9, IL6, CXCL8 | ARHGEF12, SKAP2, MAP2K6 |
| hsa05418 | Fluid shear stress and atherosclerosis | 45 | 10 | 0.2832080 | 1.4e-05 | 3.7e-05 | NPPC, MAPK9, IFNG | IL1R2, MAP3K5, MAP2K6, PDGFB, NCF2 |
| hsa04664 | Fc epsilon RI signaling pathway | 34 | 10 | 0.2068966 | 1.5e-05 | 3.6e-04 | LYN, MAPK9 | MAP2K6 |
| hsa04072 | Phospholipase D signaling pathway | 22 | 10 | 0.1403509 | 1.5e-05 | 6.0e-04 | CXCL8, F2R | EGF, PDGFB |
| hsa05416 | Viral myocarditis | 81 | 10 | 0.2261041 | 1.6e-05 | 6.7e-05 | CXADR, CASP8, BID | DAG1, CD40LG, CD28 |
| hsa04380 | Osteoclast differentiation | 43 | 10 | 0.1947055 | 2.1e-05 | 2.2e-04 | CSF1, IFNG, MAPK9 | TNFSF11, MAP2K6, NCF2, FCGR2B |
| hsa04750 | Inflammatory mediator regulation of TRP channels | 24 | 10 | 0.1565336 | 2.1e-05 | 4.2e-05 | MAPK9 | MAP2K6, PRKCQ |
| hsa05208 | Chemical carcinogenesis - reactive oxygen species | 27 | 10 | 0.1857769 | 2.2e-05 | 2.3e-04 | NDUFS6, COX5B, HGF, MAPK9 | NCF2, EGF, MAP3K5 |
| hsa04071 | Sphingolipid signaling pathway | 20 | 10 | 0.1578947 | 2.6e-05 | 6.0e-05 | BID, MAPK9 | MAP3K5 |
| hsa05166 | Human T-cell leukemia virus 1 infection | 21 | 10 | 0.1206897 | 2.7e-05 | 1.1e-04 | MAPK9, IL15, IL6 | IL1R2, TNFRSF13C, LTA |
| hsa05223 | Non-small cell lung cancer | 33 | 10 | 0.1770050 | 4.2e-05 | 7.6e-05 | HGF | EGF, RET |
| hsa05226 | Gastric cancer | 26 | 10 | 0.1896552 | 5.2e-05 | 5.2e-05 | WNT9A, HGF, FGF21, FGF23 | EGF |
| hsa05219 | Bladder cancer | 80 | 10 | 0.1565336 | 5.7e-05 | 1.2e-04 | CXCL8 | HBEGF, EGF, MMP1 |
| hsa05160 | Hepatitis C | 26 | 10 | 0.1913188 | 7.3e-05 | 5.4e-04 | IFNG, CASP8, BID | EGF, FASLG |
| hsa05210 | Colorectal cancer | 27 | 10 | 0.1403509 | 7.4e-05 | 9.9e-05 | MAPK9, AREG | EGF |
| hsa05231 | Choline metabolism in cancer | 27 | 10 | 0.1929825 | 7.4e-05 | 9.9e-05 | MAPK9 | EGF, PDGFB |
| hsa05205 | Proteoglycans in cancer | 24 | 10 | 0.0885025 | 7.4e-05 | 4.0e-04 | HGF, WNT9A | ARHGEF12, ERBB4, FASLG, HBEGF |
| hsa05322 | Systemic lupus erythematosus | 24 | 10 | 0.0869631 | 7.7e-05 | 2.4e-04 | TRIM21, IFNG | CD28, CD40LG |
| hsa05340 | Primary immunodeficiency | 45 | 10 | 0.0347852 | 7.7e-05 | 5.7e-03 |  | CD40LG, TNFRSF13C |
| hsa04640 | Hematopoietic cell lineage | 57 | 10 | 0.1724138 | 8.2e-05 | 9.2e-05 | CSF3, IL6, CSF1 | FLT3, CD1C, CD22, IL1R2 |
| hsa04920 | Adipocytokine signaling pathway | 33 | 10 | 0.0885025 | 9.1e-05 | 1.3e-04 | MAPK9, AGRP | PRKCQ |
| hsa05131 | Shigellosis | 9,422067 | 10 | 0.1217483 | 1.0e-04 | 1.7e-04 | CXCL8, MAPK9 | PRKCQ |
| hsa00514 | Other types of O-glycan biosynthesis | 5 | 10 | 0.0172414 | 1.0e-04 | 1.0e-04 |  | GALNT10, GALNT2, GALNT7 |
| hsa04066 | HIF-1 signaling pathway | 3 | 10 | 0.2087114 | 1.3e-04 | 3.3e-04 | IL6, IFNG | EGF, FLT1 |
| hsa05143 | African trypanosomiasis | 7 | 10 | 0.1565336 | 1.4e-04 | 1.5e-04 | IFNG, IL6 | FASLG |
| hsa00983 | Drug metabolism - other enzymes | 3 | 9 | 0.0172414 | 1.6e-04 | 1.6e-04 | TPMT | RRM2B, NME3 |
| hsa05320 | Autoimmune thyroid disease | 6 | 10 | 0.1217483 | 1.7e-04 | 2.9e-04 |  | CD28, FASLG, GZMB, CD40LG |
| hsa04514 | Cell adhesion molecules | 3 | 10 | 0.1052632 | 1.8e-04 | 1.8e-04 |  | CD58, CD28, ICOSLG, CD40LG, CD22, L1CAM |
| hsa05212 | Pancreatic cancer | 2 | 10 | 0.1403509 | 1.9e-04 | 7.6e-03 | MAPK9 | EGF |
| hsa05215 | Prostate cancer | 2 | 10 | 0.1578947 | 2.3e-04 | 4.1e-04 |  | IL1R2, PDGFB, EGF |
| hsa04611 | Platelet activation | 2 | 10 | 0.0517241 | 3.6e-04 | 5.1e-03 | F2R, LYN | ARHGEF12 |
| hsa04912 | GnRH signaling pathway | 3 | 10 | 0.1416040 | 4.4e-04 | 9.0e-04 | MAPK9 | MAP2K6, HBEGF |
| hsa05214 | Glioma | 2 | 10 | 0.1228070 | 4.6e-04 | 4.9e-04 |  | EGF, PDGFB |
| hsa04926 | Relaxin signaling pathway | 2 | 10 | 0.0695705 | 5.1e-04 | 9.4e-04 | MAPK9 | VEGFC, VEGFD, MMP1 |
| hsa04140 | Autophagy - animal | 1 | 10 | 0.0708020 | 7.6e-04 | 2.3e-03 | MAPK9 | PRKCQ |
| hsa04935 | Growth hormone synthesis, secretion and action | 2 | 10 | 0.1052632 | 8.1e-04 | 1.6e-03 | MAPK9 | GH2, MAP2K6 |
| hsa04218 | Cellular senescence | 1 | 10 | 0.1880479 | 9.1e-04 | 1.3e-03 | IL6, CXCL8 | MAP2K6 |
| hsa05211 | Renal cell carcinoma | 2 | 10 | 0.1206897 | 1.1e-03 | 8.2e-03 | HGF | PDGFB |
| hsa05017 | Spinocerebellar ataxia | 1 | 10 | 0.0862069 | 1.1e-03 | 2.3e-03 | MAPK9 | MAP3K5 |
| hsa05230 | Central carbon metabolism in cancer | 2 | 10 | 0.0344828 | 1.2e-03 | 1.4e-03 |  | RET. FLT3 |
| hsa05202 | Transcriptional misregulation in cancer | 3 | 10 | 0.1724138 | 1.4e-03 | 1.7e-03 | IL6, CXCL8 | GZMB, FLT3, BIRC2, IL1R2, FLT1 |
| hsa05415 | Diabetic cardiomyopathy | 2 | 10 | 0.0526478 | 1.5e-03 | 3.2e-02 | MAPK9, NDUFS6, COX5B | NCF2 |
| hsa04917 | Prolactin signaling pathway | 2 | 10 | 0.0521779 | 1.5e-03 | 6.8e-03 | MAPK9 | TNFSF11 |
| hsa04662 | B cell receptor signaling pathway | 3 | 10 | 0.1538574 | 1.5e-03 | 1.5e-03 | LYN | FCGR2B, CD22 |
| hsa04530 | Tight junction | 1 | 10 | 0.0689655 | 1.6e-03 | 1.7e-03 | MAPK9 | CD1C, MAP3K5 |
| hsa04141 | Protein processing in endoplasmic reticulum | 2 | 10 | 0.0689655 | 1.7e-03 | 3.6e-03 | CKAP4, PREB, MAPK9 | DNAJA2, MAP3K5 |
| hsa04540 | Gap junction | 2 | 10 | 0.1228070 | 1.7e-03 | 2.3e-03 |  | PDGFB, EGF |
| hsa05203 | Viral carcinogenesis | 0.7569061 | 10 | 0.0695705 | 1.8e-03 | 5.8e-03 | LYN, CASP8 |  |
| hsa05020 | Prion disease | 1 | 10 | 0.0766979 | 2.4e-03 | 1.2e-02 | NDUFS6, COX5B, MAPK9, IL6 | NCF2 |
| hsa04666 | Fc gamma R-mediated phagocytosis | 2 | 10 | 0.1025716 | 2.5e-03 | 2.5e-03 | LYN | FCGR2B |
| hsa05412 | Arrhythmogenic right ventricular cardiomyopathy | 2 | 10 | 0.0175439 | 3.0e-03 | 4.0e-03 |  | ITGB6, DAG1 |
| hsa05310 | Asthma | 3 | 10 | 0.0173926 | 3.2e-03 | 4.3e-03 |  | CD40LG |
| hsa04623 | Cytosolic DNA-sensing pathway | 3 | 10 | 0.0173926 | 3.2e-03 | 5.5e-03 | POLR2F, IL6 |  |
| hsa04512 | ECM-receptor interaction | 3 | 10 | 0.0350877 | 3.4e-03 | 4.3e-03 |  | COL9A1, ITGB6, DAG1 |
| hsa05410 | Hypertrophic cardiomyopathy | 3 | 10 | 0.0695705 | 3.6e-03 | 4.4e-03 | IL6 | ITGB6, DAG1 |
| hsa04930 | Type II diabetes mellitus | 2 | 10 | 0.0173926 | 3.7e-03 | 6.1e-03 | MAPK9 |  |
| hsa05414 | Dilated cardiomyopathy | 2 | 10 | 0.0175439 | 3.7e-03 | 4.6e-03 |  | ITGB6, DAG1 |
| hsa05012 | Parkinson disease | 2 | 10 | 0.0347852 | 4.4e-03 | 9.1e-03 | SLC18A1, SLC39A5, NDUFS6, COX5B, MAPK9 | MAP3K5 |
| hsa05140 | Leishmaniasis | 2 | 10 | 0.0708020 | 5.2e-03 | 7.3e-03 | IFNG | NCF2 |
| hsa00480 | Glutathione metabolism | 6 | 8 | 0.0512858 | 5.7e-03 | 2.2e-02 | LAP3, PRDX6 | HPGDS, RRM2B |
| hsa04137 | Mitophagy - animal | 1 | 10 | 0.0695705 | 5.8e-03 | 7.3e-03 | MAPK9 |  |
| hsa04360 | Axon guidance | 2 | 9 | 0.0178571 | 5.8e-03 | 5.8e-03 |  | ARHGEF12, PLXNA4, L1CAM, SEMA4C |
| hsa05213 | Endometrial cancer | 1 | 10 | 0.0701754 | 6.1e-03 | 6.9e-03 |  | EGF |
| hsa05216 | Thyroid cancer | 2 | 10 | 0.0344828 | 7.0e-03 | 8.7e-03 |  | RET |
| hsa04730 | Long-term depression | 3 | 10 | 0.0338983 | 8.4e-03 | 1.9e-02 | LYN | CRH |
| hsa04024 | cAMP signaling pathway | 1 | 10 | 0.0347852 | 8.6e-03 | 4.2e-02 | MAPK9, F2R | CRH |
| hsa05225 | Hepatocellular carcinoma | 0.9 | 10 | 0.0341905 | 8.8e-03 | 2.9e-02 | WNT9A, HGF |  |
| hsa04310 | Wnt signaling pathway | 1 | 10 | 0.0683811 | 8.8e-03 | 2.7e-02 | WNT9A, RSPO3, MAPK9 | |
| hsa04914 | Progesterone-mediated oocyte maturation | 0.8 | 10 | 0.0172414 | 1.1e-02 | 1.7e-02 | MAPK9 |  |
| hsa04670 | Leukocyte transendothelial migration | 0.7 | 10 | 0.0517241 | 1.1e-02 | 1.6e-02 |  | NCF2 |
| hsa05207 | Chemical carcinogenesis - receptor activation | 0.8 | 10 | 0.0175439 | 1.1e-02 | 3.2e-02 |  | TNFSF11, EGF |
| hsa05221 | Acute myeloid leukemia | 1 | 10 | 0.0344828 | 1.3e-02 | 2.0e-02 |  | FLT3 |
| hsa00190 | Oxidative phosphorylation | 1 | 10 | 0.0172414 | 1.3e-02 | 1.3e-02 | NDUFS6, COX5B |  |
| hsa04910 | Insulin signaling pathway | 0.5 | 10 | 0.0344828 | 1.3e-02 | 1.3e-02 | MAPK9 |  |
| hsa04928 | Parathyroid hormone synthesis, secretion and action | 2 | 10 | 0.0517241 | 1.5e-02 | 2.3e-02 | FGF23 | TNFSF11, HBEGF |
| hsa04612 | Antigen processing and presentation | 1 | 10 | 0.0344828 | 1.5e-02 | 1.5e-02 | IFNG |  |
| hsa04270 | Vascular smooth muscle contraction | 2 | 10 | 0.0177005 | 1.6e-02 | 3.3e-02 | ADM, NPPC | PRKCQ, ARHGEF12 |
| hsa04919 | Thyroid hormone signaling pathway | 0.6 | 10 | 0.0172414 | 1.8e-02 | 1.9e-02 |  | PFKFB2 |
| hsa04152 | AMPK signaling pathway | 0.6 | 10 | 0.0172414 | 1.8e-02 | 1.9e-02 |  | PFKFB2 |
| hsa04728 | Dopaminergic synapse | 1 | 10 | 0.0172414 | 1.8e-02 | 3.0e-02 | SLC18A1, MAPK9 |  |
| hsa04723 | Retrograde endocannabinoid signaling | 1 | 10 | 0.0172414 | 2.0e-02 | 3.3e-02 | MAPK9, NDUFS6 |  |
| hsa04120 | Ubiquitin mediated proteolysis | 0.5 | 10 | 0.0341905 | 2.1e-02 | 2.1e-02 |  | BIRC2 |
| hsa05150 | Staphylococcus aureus infection | 2 | 10 | 0.0172414 | 2.2e-02 | 2.2e-02 | KRT18 | FCGR2B |
| hsa04613 | Neutrophil extracellular trap formation | 0.4 | 10 | 0.0512858 | 2.2e-02 | 3.2e-02 |  | NCF2 |
| hsa00380 | Tryptophan metabolism | 2 | 8 | 0.0170953 | 2.2e-02 | 2.2e-02 |  | KYNU |
| hsa05222 | Small cell lung cancer | 0.8 | 10 | 0.0172414 | 2.4e-02 | 2.5e-02 |  | BIRC2 |
| hsa05217 | Basal cell carcinoma | 1 | 9 | 0.0172414 | 2.5e-02 | 2.5e-02 | WNT9A |  |
| hsa04915 | Estrogen signaling pathway | 1 | 3 | 0.0491803 | 2.8e-02 | 4.9e-02 | KRT18 | HBEGF |
| hsa00562 | Inositol phosphate metabolism | 1 | 9 | 0.0172414 | 3.4e-02 | 3.4e-02 | INPP1 |  |
| hsa04070 | Phosphatidylinositol signaling system | 0.8 | 9 | 0.0172414 | 3.4e-02 | 3.4e-02 | INPP1 |  |
| hsa04350 | TGF-beta signaling pathway | 2 | 9 | 0.0172414 | 3.5e-02 | 3.5e-02 | FST, IFNG |  |
| hsa04916 | Melanogenesis | 0.7 | 9 | 0.0172414 | 4.1e-02 | 4.1e-02 | WNT9A |  |
| hsa04714 | Thermogenesis | 1 | 10 | 0.0172414 | 4.3e-02 | 4.3e-02 | FGF21, NDUFS6, COX5B | MAP3K5 |
| hsa04145 | Phagosome | 2 | 10 | 0.0172414 | 4.8e-02 | 4.8e-02 |  | FCGR2B, SFTPA2, NCF2 |

**Supplementary Table 5.** GO enriched terms for the 202 DEPs between CRC patients and healthy controls.

| ID | Description | setSize | enrichmentScore | NES | pvalue | p.adjust | qvalue | rank | leading_edge | core_enrichment |
| --- | --- | --- | --- | --- | --- | --- | --- | --- | --- | --- |
| GO:0043086 | negative regulation of catalytic activity | 14 | 0.720 | 2.152 | 0.0001 | 0.048 | 0.047 | 40 | tags=71%. list=20%. signal=62% | 93974/2281/51400/3458/51129/9270/51499/4067/5806/27290 |
| GO:0042391 | regulation of membrane potential | 4 | 0.935 | 1.837 | 0.0001 | 0.048 | 0.047 | 6 | tags=75%. list=3%. signal=74% | 637/93974/2281 |
| GO:0006119 | oxidative phosphorylation | 3 | 0.960 | 1.700 | 0.0002 | 0.048 | 0.047 | 11 | tags=100%. list=5%. signal=96% | 637/4726/1329 |
| GO:0009060 | aerobic respiration | 3 | 0.960 | 1.700 | 0.0002 | 0.048 | 0.047 | 11 | tags=100%. list=5%. signal=96% | 637/4726/1329 |
| GO:0022904 | respiratory electron transport chain | 3 | 0.960 | 1.700 | 0.0002 | 0.048 | 0.047 | 11 | tags=100%. list=5%. signal=96% | 637/4726/1329 |
| GO:0042773 | ATP synthesis coupled electron transport | 3 | 0.960 | 1.700 | 0.0002 | 0.048 | 0.047 | 11 | tags=100%. list=5%. signal=96% | 637/4726/1329 |
| GO:0042775 | mitochondrial ATP synthesis coupled electron transport | 3 | 0.960 | 1.700 | 0.0002 | 0.048 | 0.047 | 11 | tags=100%. list=5%. signal=96% | 637/4726/1329 |

**Supplementary Table 6.** DEPs between CRC patients and healthy controls that are identified in the human blood secretome from Human Protein Atlas.

| Secretome proteins | |
| --- | --- |
| ADM | IL16 |
| ANGPTL4 | IL17A |
| CCL20 | IL1R2 |
| CCL23 | IL6 |
| CD40LG | LGALS9 |
| CREG1 | LTA |
| CRH | MDK |
| CSF1 | MLN |
| CSF3 | MZB1 |
| CXCL8 | NELL2 |
| CXCL9 | NPPC |
| ESM1 | NUCB2 |
| FASLG | PDGFB |
| FGF21 | PON3 |
| FGF23 | PTX3 |
| FLT1 | RSPO3 |
| FST | S100A12 |
| GH2 | SIGLEC6 |
| GZMB | SMPDL3A |
| HBEGF | SPARC |
| HGF | SPON1 |
| IFNG | TNFSF10 |
| IL15 | TNFSF11 |
| VEGFC | TNFSF12 |
| VEGFD | TPSAB1 |

**Supplementary Table 7.** Proteins significantly correlated with inflammation status with the corresponding correlation coefficient and *p*-value.

| Protein | Coefficient | p-value |
| --- | --- | --- |
| DCXR | -0.500 | 0.001 |
| SORD | -0.487 | 0.002 |
| PVALB | -0.458 | 0.004 |
| IL12RB1 | 0.451 | 0.005 |
| PSPN | -0.447 | 0.005 |
| SHMT1 | -0.444 | 0.005 |
| RBP5 | -0.440 | 0.006 |
| CXCL6 | -0.433 | 0.007 |
| IL1R2 | -0.431 | 0.007 |
| APBB1IP | -0.426 | 0.008 |
| PLXNA4 | -0.401 | 0.013 |
| ATG4A | -0.395 | 0.014 |
| CA11 | 0.394 | 0.014 |
| MGMT | -0.394 | 0.014 |
| DNAJB1 | -0.392 | 0.015 |
| KYNU | -0.386 | 0.017 |
| GLOD4 | -0.385 | 0.017 |
| CCL13 | -0.381 | 0.018 |
| TRAF2 | -0.378 | 0.019 |
| AIFM1 | -0.376 | 0.020 |
| DDAH1 | -0.375 | 0.020 |
| NUDT2 | -0.375 | 0.020 |
| CSF3 | 0.374 | 0.021 |
| DNAJA2 | -0.374 | 0.021 |
| ALDH3A1 | -0.367 | 0.023 |
| DCTN1 | -0.365 | 0.024 |
| HGS | -0.364 | 0.025 |
| ACP6 | -0.364 | 0.025 |
| CASP8 | -0.362 | 0.026 |
| CD276 | 0.358 | 0.027 |
| ACAA1 | -0.354 | 0.029 |
| NT5C3A | -0.350 | 0.031 |
| FXN | -0.350 | 0.031 |
| CPE | -0.349 | 0.032 |
| GMPR | -0.349 | 0.032 |
| VPS37A | -0.347 | 0.033 |
| PROK1 | -0.347 | 0.033 |
| DNER | -0.345 | 0.034 |
| COX5B | -0.345 | 0.034 |
| WIF1 | -0.342 | 0.036 |
| LHPP | -0.341 | 0.036 |
| METAP2 | -0.341 | 0.036 |
| IL3RA | 0.336 | 0.039 |
| ADAMTS15 | -0.335 | 0.040 |
| S100A4 | -0.333 | 0.041 |
| CIAPIN1 | -0.331 | 0.042 |
| SCLY | -0.331 | 0.043 |
| MILR1 | 0.329 | 0.044 |
| AMIGO2 | -0.327 | 0.045 |
| DNPH1 | -0.326 | 0.046 |
| MYO9B | -0.325 | 0.046 |
| SEMA4C | 0.323 | 0.048 |
| ATP5IF1 | -0.323 | 0.048 |
| P4HB | -0.321 | 0.049 |
| RNF41 | -0.321 | 0.049 |
| TRIM21 | -0.321 | 0.050 |

**Supplementary Table 8.** DEPs between patients with and without inflammation. The FC is defined as patients with inflammation - patients without inflammation (Inf-NonInf).

| Protein | logFC(Inf-NonInf) | adj.p.value |
| --- | --- | --- |
| PSPN | -1.377 | 0.002 |
| DCXR | -0.870 | 0.004 |
| SORD | -1.440 | 0.005 |
| PVALB | -1.637 | 0.010 |
| IL12RB1 | 0.477 | 0.010 |
| IL1R2 | -0.262 | 0.010 |
| CXCL6 | -1.059 | 0.012 |
| SHMT1 | -1.468 | 0.014 |
| APBB1IP | -0.561 | 0.015 |
| RBP5 | -0.598 | 0.016 |
| ACP6 | -0.568 | 0.020 |
| ATG4A | -1.122 | 0.030 |
| CA11 | 0.354 | 0.032 |
| PLXNA4 | -1.049 | 0.032 |
| CCL13 | -0.751 | 0.034 |
| KYNU | -0.652 | 0.037 |
| DNAJB1 | -1.635 | 0.037 |
| MGMT | -1.424 | 0.038 |
| ALDH3A1 | -0.962 | 0.043 |
| CSF3 | 0.605 | 0.043 |
| GLOD4 | -0.648 | 0.044 |
| TRAF2 | -0.930 | 0.045 |
| PROK1 | -0.573 | 0.046 |
| CD276 | 0.425 | 0.046 |
| NUDT2 | -0.827 | 0.048 |
| DDAH1 | -0.430 | 0.048 |

**Supplementary Table 9.** KEGG enriched terms for the 26 DEPs between CRC patients with and without inflammation. Each term contains an associated description, the fold enrichment, the occurrence, the support, the lowest/highest p-value in the iterations, as well as the up/down-regulated proteins.

| ID | Term_Description | Fold_Enrichment | occurrence | support | lowest_p | highest_p | Up_regulated | Down_regulated |
| --- | --- | --- | --- | --- | --- | --- | --- | --- |
| hsa04060 | Cytokine-cytokine receptor interaction | 10 | 10 | 0.3 | 6.0e-05 | 6.0e-05 | CSF3, IL12RB1 | CCL13, CXCL6, IL1R2 |
| hsa04657 | IL-17 signaling pathway | 18 | 10 | 0.3 | 9.4e-05 | 9.4e-05 | CSF3 | TRAF2, CXCL6 |
| hsa04630 | JAK-STAT signaling pathway | 7 | 10 | 0.2 | 6.2e-04 | 6.2e-04 | CSF3, IL12RB1 | |
| hsa00040 | Pentose and glucuronate interconversions | 37 | 10 | 0.1 | 1.5e-03 | 1.5e-03 |  | DCXR, SORD |
| hsa00410 | beta-Alanine metabolism | 18 | 10 | 0.1 | 4.6e-03 | 4.6e-03 |  | ALDH3A1 |
| hsa05144 | Malaria | 12 | 10 | 0.3 | 5.7e-03 | 5.7e-03 | CSF3 |  |
| hsa04622 | RIG-I-like receptor signaling pathway | 8 | 10 | 0.2 | 5.7e-03 | 5.7e-03 |  | TRAF2 |
| hsa04920 | Adipocytokine signaling pathway | 8 | 10 | 0.2 | 5.7e-03 | 5.7e-03 |  | TRAF2 |
| hsa05321 | Inflammatory bowel disease | 9 | 10 | 0.2 | 7.3e-03 | 7.3e-03 | IL12RB1 |  |
| hsa04658 | Th1 and Th2 cell differentiation | 6 | 10 | 0.1 | 7.6e-03 | 7.6e-03 | IL12RB1 |  |
| hsa05222 | Small cell lung cancer | 6 | 10 | 0.1 | 7.6e-03 | 7.6e-03 |  | TRAF2 |
| hsa04064 | NF-kappa B signaling pathway | 1 | 10 | 0.2 | 7.6e-03 | 7.6e-03 |  | TRAF2, CCL13 |
| hsa04659 | Th17 cell differentiation | 5 | 10 | 0.2 | 7.6e-03 | 7.6e-03 | IL12RB1 |  |
| hsa04668 | TNF signaling pathway | 10 | 10 | 0.3 | 7.6e-03 | 7.6e-03 |  | TRAF2, CXCL6 |
| hsa04380 | Osteoclast differentiation | 5 | 10 | 0.3 | 7.9e-03 | 7.9e-03 |  | TRAF2 |
| hsa04210 | Apoptosis | 4 | 10 | 0.2 | 7.9e-03 | 7.9e-03 |  | TRAF2 |
| hsa05135 | Yersinia infection | 4 | 10 | 0.2 | 7.9e-03 | 7.9e-03 |  | TRAF2 |
| hsa04640 | Hematopoietic cell lineage | 12 | 10 | 0.3 | 7.9e-03 | 7.9e-03 | CSF3 | IL1R2 |
| hsa04071 | Sphingolipid signaling pathway | 5 | 10 | 0.2 | 7.9e-03 | 7.9e-03 |  | TRAF2 |
| hsa04932 | Non-alcoholic fatty liver disease | 4 | 10 | 0.1 | 8.3e-03 | 8.3e-03 |  | TRAF2 |
| hsa05160 | Hepatitis C | 4 | 10 | 0.2 | 8.3e-03 | 8.3e-03 |  | TRAF2 |
| hsa00380 | Tryptophan metabolism | 14 | 10 | 0.1 | 8.6e-03 | 8.6e-03 |  | KYNU |
| hsa04217 | Necroptosis | 4 | 10 | 0.1 | 8.7e-03 | 8.7e-03 |  | TRAF2 |
| hsa05133 | Pertussis | 8 | 10 | 0.1 | 8.9e-03 | 8.9e-03 |  | CXCL6 |
| hsa04621 | NOD-like receptor signaling pathway | 3 | 10 | 0.2 | 9.8e-03 | 9.8e-03 |  | TRAF2 |
| hsa05130 | Pathogenic Escherichia coli infection | 3 | 10 | 0.3 | 9.8e-03 | 9.8e-03 |  | TRAF2 |
| hsa05323 | Rheumatoid arthritis | 7 | 10 | 0.1 | 1.0e-02 | 1.0e-02 |  | CXCL6 |
| hsa04061 | Viral protein interaction with cytokine and cytokine receptor | 12 | 10 | 0.1 | 1.0e-02 | 1.0e-02 |  | CCL13, CXCL6 |
| hsa05169 | Epstein-Barr virus infection | 3 | 10 | 0.2 | 1.1e-02 | 1.1e-02 |  | TRAF2 |
| hsa05170 | Human immunodeficiency virus 1 infection | 3 | 10 | 0.2 | 1.1e-02 | 1.1e-02 |  | TRAF2 |
| hsa05417 | Lipid and atherosclerosis | 3 | 10 | 0.2 | 1.1e-02 | 1.1e-02 |  | TRAF2 |
| hsa05163 | Human cytomegalovirus infection | 3 | 10 | 0.2 | 1.1e-02 | 1.1e-02 |  | TRAF2 |
| hsa05171 | Coronavirus disease - COVID-19 | 3 | 10 | 0.2 | 1.1e-02 | 1.1e-02 | CSF3 |  |
| hsa05167 | Kaposi sarcoma-associated herpesvirus infection | 3 | 10 | 0.1 | 1.1e-02 | 1.1e-02 |  | TRAF2 |
| hsa05203 | Viral carcinogenesis | 3 | 10 | 0.1 | 1.1e-02 | 1.1e-02 |  | TRAF2 |
| hsa05131 | Shigellosis | 2 | 10 | 0.2 | 1.3e-02 | 1.3e-02 |  | TRAF2 |
| hsa05132 | Salmonella infection | 2 | 10 | 0.2 | 1.3e-02 | 1.3e-02 |  | TRAF2 |
| hsa05146 | Amoebiasis | 6 | 10 | 0.1 | 1.5e-02 | 1.5e-02 |  | IL1R2 |

**Supplementary Table 10.** Proteins significantly correlated with cancer stage with the corresponding correlation coefficient and *p*-value.

| Protein | Coefficient | p-value |
| --- | --- | --- |
| IL32 | -0.464 | 0.003 |
| TMPRSS15 | -0.385 | 0.017 |
| IFNG | -0.367 | 0.023 |
| SELPLG | -0.356 | 0.028 |
| TFF2 | -0.354 | 0.029 |
| ITGAV | -0.337 | 0.039 |
| ITGA11 | -0.328 | 0.044 |
| S100A12 | 0.328 | 0.045 |
| MZB1 | 0.333 | 0.041 |
| IL6 | 0.341 | 0.036 |
| IL1R2 | 0.345 | 0.034 |
| WFIKKN2 | 0.353 | 0.029 |
| SMOC2 | 0.362 | 0.025 |
| ACP6 | 0.364 | 0.024 |
| CCL23 | 0.370 | 0.022 |
| TXNDC15 | 0.388 | 0.016 |
| SCGB1A1 | 0.404 | 0.012 |
| CLEC4G | 0.406 | 0.011 |
| MANSC1 | 0.448 | 0.005 |
| FLT4 | 0.482 | 0.002 |

**Supplementary Table 11.** DEPs between early and late-stage patients. The FC is defined as patients with late-stage CRC - patients with early-stage CRC.

| Protein | logFC(Late-Early) | adj.p.value |
| --- | --- | --- |
| IFNG | -1.124 | 0.048 |
| IL32 | -0.427 | 0.017 |
| FLT4 | 0.370 | 0.011 |
| MANSC1 | 0.261 | 0.015 |
| ACP6 | 0.548 | 0.020 |
| IL17C | -0.539 | 0.039 |
